# Supplementary material for: The 3D architecture of the pepper genome and its relationship to function and evolution
Source: Nat Commun. 2022 Jun 16;13:3479. doi: 10.1038/s41467-022-31112-x (PMC9203530; doi:10.1038/s41467-022-31112-x)
Supplement: Supplementary file 1 — Supplementary Information [file 41467_2022_31112_MOESM1_ESM.pdf]

The 3D architecture of the pepper genome and its relationship to function and evolution

Liao *et al*.

Supplementary Method 1. Genome size estimation

To obtain preliminary information on the genomic characteristics before large-scale genome sequencing for the inbred line CA59, we first used G.C.E (Genome Characteristics Estimation)(1.0.2)[1] with ~362.0 Gb BGI short reads (150 bp pair-end) to estimate the genome size, repeat content, and heterozygous rate based on the distribution of 17-mer frequency calculated by *kmerfreq*. Quality control of BGI sequencing short reads was conducted using Trimmomatic (0.38)[2].

The Kmer_number is calculated using the following formula:

Kmer_number = reads_number * (reads_len - kmer_len + 1). (**1**)

The Heterozygous rate and repeat frequency are calculated using the following formulas:

Heterozygous rate = a[½] / (2 - a[½]) (**2**)

Repeat frequency = 1 - b[½] - b[1] (**3**)

where a[½] indicates the ratio of unique k-mers in all the kmer species in the half genome coverage peak of genome coverage peak, b[1] indicates the ratio of unique k-mers in all the kmer individuals in the genome, and b[½] indicates the ratio of unique k-mers in all the k-mers individuals in the half genome coverage peak of genome coverage peak.

The parameters of *kmerfreq* and G.C.E were set as follows:

```
kmerfreq -k 17 -t 48 -r 10000 -p Pepper_survey reads_list_file
less ${prefix}.kmer.freq.stat | perl -ne 'next if(/^#/ || /^\s/); print; ' | awk '{print $1"\\t"$2}' > ${prefix}.kmer.freq.stat.2colum
gce -f ${prefix}.kmer.freq.stat.2colum -g ${kmer_number} -m 1 -D 8 -b 0 -H 1 -c ${cov} 1> ${prefix}.table 2 > ${prefix}.gce.result
```

The G.C.E analysis shows that the estimated genome size of the CA59 line is about 2.95 Gb, the heterozygous rate is about 0.23%, and the repeat frequency of the genome is about 76.17%.

Supplementary Method 2. Genome assembly

De novo assembly of the CA59 genome was carried out as follows:

Step 1: Long reads (PacBio) sequencing

Extraction of high-molecular-weight DNA from young leaves was carried out using a modified cetyltrimethylammonium bromide (CTAB) method[3]. About 10 μg of genomic DNA was used for preparing template libraries of 30~40-kb using the BluePippin Size Selection system (Sage Science, USA) following the manufacturer’s protocol (Pacific Biosciences, USA). The libraries were sequenced

on the PacBio SEQUEL II platform with three SMRT flow cells. The summary statistics of raw PacBio long reads are provided in Supplementary Table 1.

Step 2: Filtering out short and low-quality reads

To filter out short and low-quality raw PacBio long reads, we sorted all reads based on their length in descending order. We only included the top 200.0 Gb longest reads for genome assembling. The summary statistics of the selected PacBio long reads for genome assembly are provided in Supplementary Table 1.

Step 3: Correction

Next, we used MECAT2 (v20220228)[4] to correct the selected raw PacBio long reads with the config file provided in https://github.com/yiliao1022/Pepper3Dgenome/Data_Processing/Walkthrough.sh. The command used for the correction process is

```
mecat.pl correct Ca_59.config
```

Step 4: Trimming and assembling

After correction, we used CANU (2.0)[5] to trim the corrected sequences obtained above and assemble the resulting trimmed sequences. The parameters of CANU and commands used are:

```
canu -trim-assemble -p Capsicum -d Capsicum GenomeSize=3000m corMhapFilterThreshold=0.0000000002 corMhapOptions="""--threshold 0.80 --num-hashes 512 --num-min-matches 3 --ordered-sketch-size 1000 --ordered-kmer-size 17 --min-olap-length 2000 --repeat-idf-scale 50""" mhapBlockSize=500 ovlMerThreshold=500 minReadLength=30000 minOverlapLength=2000  -pacbio-corrected cns_final.fasta &>>canu.log
```

Step 5: Polishing using short reads

The assembled contigs were further polished with ~123 depth of BGI short reads data using Pilon (1.23)[6]. Three rounds of the polishing run were performed iteratively on the CANU assembly. The parameters of Pilon and commands used are:

```
pilon --threads ${threads} --genome ${draft_genome} --frags ${mapped_bam} --fix snps,indels --output ${polished_genome}
```

Step 6: Quality evaluation

To assess the quality of the genome assembly, we calculated two metrics: BUSCO and the Phred quality score QV value. We used BUSCO (3.02)[7] based on the embryophyta_odb9 data set to assess the completeness of the gene space of the assembly. We mapped BGI short reads to the final polished

assembly using Bowtie2 (2.4.4)[8] with the default parameters. Freebayes (1.3.4)[9] was run with the command:

```
freebayes -C 2 -0 -O -q 20 -z 0.10 -E 0 -X -u -p 2 -F 0.75 -b QV_mapping.bam -v QV.vcf -f Capsicum_finalpolsih.fasta
```

The QV was computed as

$QV = -10\log_{10}(B/T)$,[4]

where B was the total number of variant sites (insertions/deletions/SNPs) obtained from the above QV.vcf file, and T is the number of the genome sites with at least 3 mapped reads..

BUSCO was run with the command:

```
run_BUSCO.py -i ${genome_file} -l Busco_database/embryophyta_odb9 -o ${genome_file}.checkresult -m genome -c ${threads} -f
```

Step 7: Scaffolding

Finally, we used the Juicer/Juicerbox/3D-DNA (version 180114)[10,11] workflow with a combination of Hi-C data from two tissues including flower bud and leaf, totaling 415.2 Gb, corresponding to ~135 depth of genome coverage, to scaffold the contigs. The Juicer and 3D-DNA were run with the commands: Juicer:

```
juicer.sh -g contig_ -d `pwd` -s MboI -z polished_contigs.fa -t 40 -y hic_MboI.txt -p polished_contigs.fa.size
```

3D-DNA:

```
3d-dna/run-asm-pipeline.sh -r 0 ../polished_contigs.fa ../aligned/merged_nodups.txt
```

Supplementary Method 3. ISO-Seq full-length transcriptome data processing

We used SMRTlink (version 8) (https://www.pacb.com/support/software-downloads/), to process the subreads to FLNC (Full-Length non-chimericRead) reads. The TAMA (c090ae)[12] pipeline (run in python version 2.7.17 environment) was used to remove redundant alignments in *.bam* files, according to PacBio's official recommendation. Next, the Ucsc-bedToGenePred and ucsc-genePredToGtf (377)[13] were used to convert the resulting *.bam* file to .gtf file. Then, the Gffread[14] was used to extract mRNA sequences from the genome assembly. TransDecoder (5.5.0) (https://github.com/TransDecoder/TransDecoder) was used to predict coding sequences and peptide sequences from mRNA sequences. The SMRTlink pipeline was run with the commands:

```
ccs ${prefix}.subreads.bam ${prefix}.ccs.bam --noPolish --minPasses 1
lima ${prefix}.ccs.bam primers.fa ${prefix}.demux.ccs.bam --isoseq --peek-guess
isoseq3 refine --require-polya ${prefix}.demux.ccs.F1_5p--R1_3p.bam primers.fa ${prefix}.flnc.bam
```

Supplementary Method 4. RNA-seq protocol

Total RNA was extracted using Trizol reagent following the manufacturer's recommendations (Invitrogen, CA, USA). RNA purity and integrity were assessed using NanoDrop 2000 spectrophotometer (NanoDrop Technologies, Wilmington, DE, USA) and Bioanalyzer 2100 system (Agilent Technologies, CA, USA). RNA contamination was assessed using 1.5% agarose gel electrophoresis. A total of 1 μg of RNA per sample was used as the input material for library preparation. The mRNA was purified from the total RNA using poly- T oligo- attached magnetic beads. Sequencing libraries were generated from the purified mRNA using the V AHTS Universal V6 RNA-seq Library Kit for MGI (Vazyme, Nanjing, China) following the manufacturer's recommendations with unique index codes. The size of the resulting library was assessed using Qubit 3.0 Fluorometer (Life Technologies, Carlsbad, CA, USA) and Bioanalyzer 2100 system (Agilent Technologies, CA, USA). Subsequently, sequencing was performed on the MGI-SEQ 2000 platform by Frasergen Bioinformatics Co., Ltd. (Wuhan, China).

Supplementary Method 5. Short-read RNA-seq data processing

We generated RNA-seq data for 5 tissues, including bud, leave, placenta, pulp, and root. Raw data were preprocessed using Trimmomatic (0.38)[2] to trim adapter sequences and filter out low-quality reads. Clean RNA-seq reads were aligned to the CA59 genome using HISAT2(2.21)[15]. StringTie (2.1.4) [16] was used to reconstruct the transcriptome based on the Maker annotation and produce the *.gtf* file. The expression level for each gene and/or transcript was quantified in normalized TPM (Transcript Per Million) and FPKM (Reads Per Kilobase of transcript per Million reads mapped) values using FeatureCounts[17] and a custom R script. Hisat2 and Stringtie were run with the commands:

```
hisat2 --dta --rg-id hisat2 --rg SM:${samplename} --threads ${threads}  -x tmp/tmpidx -1 ${reads_R1} -2 ${reads_R2} | samtools view -Shb - > hisat2/${samplename}.unsort.bam
samtools sort -@ ${threads} hisat2/${samplename}.unsort.bam > hisat2/${samplename}.sorted.bam
stringtie hisat2/${samplename}.sorted.bam  -p ${threads} -o stringtie/${samplename}.gtf -A stringtie/${samplename}.tab
```

Supplementary Method 6. TE annotation and analysis

The repeat sequence library was built by EDTA (1.9.6)[18]. The plot of Kimura distance among pairwise alignments between TE sequences identified from RepeatMasker was conducted as follows: The complete LTR sequence (Built by EDTA) was used as a repeats lib to run RepeatMasker. The script "*calcDivergenceFromAlign.pl*" from RepeatMasker was used to calculate the divergence distance of all compared TE sequence pairs. Then, the R scripts from KristinaGagalova and CraigMichell's GitHub

repository: https://github.com/oushujun/EDTA/issues/92 were used to plot the distribution of the Kimura distance. EDTA was run with the command:

```
EDTA.pl -specie others -threads $threads -overwrite 1 -genome ${genome_file}
```

Supplementary Method 7. Gene annotation

We used the MAKER pipeline[19] to annotate gene models. MAKER was run in three iterations, with each using the command: `mpiexec -n ${threads} maker -fix_nucleotides`

The config files for each run are provided in https://github.com/yiliao1022/Pepper3Dgenome/Data_Processing/.

Supplementary Method 8. Hi-C libraries construction

About 2 g of plant material was cut into 1 to 2 mm strips, which were fixed with 2% final concentration fresh formaldehyde in NIB buffer (20 mM HEPES, pH 8.0, 250 mM sucrose, 1 mM MgCl2,5mM KCl, 40% (v/v) glycerol, 0.25% (v/v) Triton X-100, 0.1 mM PMSF, and 0.1% (v/v) β-mercaptoethanol) at 4°C for 45 min in a vacuum. Formaldehyde was added at a final concentration of 0.375 M glycine under vacuum infiltration for an additional 5 min. The samples were washed twice in ice-cold water. The clean samples were frozen in liquid nitrogen and then ground to a powder and resuspended in the NIB buffer. The solution was then filtered through one layer of Miracloth. The nuclei isolated from these tissues were lysed with 0.1% (w/v) final concentration SDS at 65°C for 10 min and then SDS molecules were added using Triton X-100 at a 1% (v/v) final concentration. The DNA in the nuclei was then digested by adding 200U MboI (NEB) and incubating the samples at 37°C for 2 hr. Restriction fragment ends were labeled with biotinylated cytosine nucleotides by biotin-14-dCTP (TriLINK). Blunt-end ligation was carried out at 16°C overnight in the presence of 50 Weiss units of T4 DNA ligase. After ligation, the cross-linking was reversed by 200 µg/mL proteinase K (Thermo) at 65°C overnight. DNA purification was achieved through QIAamp DNA Mini Kit (Qiagen) according to the manufacturer's instructions. Purified DNA was sheared to a length of ~400 bp. Point ligation junctions were pulled down by Dynabeads® MyOne™ Streptavidin C1 (Thermofisher) according to the manufacturer's instructions. The Hi-C library for short reads sequencing was prepared using the VAHTS Universal Plus DNA Library Prep Kit for MGI (Vazyme, NDM617) according to the manufacturer’s instructions. Fragments between 400 and 600 bp were paired-end sequenced on the MGI-seq 2000 platform at 150 PE mode. Samples were prepared and sequenced with assistance from Frasergen Bioinformatics Co., Ltd (Wuhan, China).

Supplementary Method 9. Hi-C data processing

The raw Hi-C data were processed using two pipelines HiCExplorer (3.5.3)[20] and Juicer (1.56)[11]. They were run with the following commands:

Juicer:

```
juicer.sh -g scaffold -d `pwd` -s MboI -z assembled_genome.fa -t 40 -y hic_MboI.txt -p assembled_genome.fa.size
```

HiCexplorer:

```
bwa mem -t 24 -A1 -B4 -E50 -L0 $reference_fasta ${prefix}_mapping/${prefix}_R1.fastq.gz 2> ${prefix}_mapping/${prefix}_R1.log | samtools view -Shb - > ${prefix}_mapping/${prefix}_R1.bam

bwa mem -t 24 -A1 -B4 -E50 -L0 $reference_fasta ${prefix}_mapping/${prefix}_R2.fastq.gz 2> ${prefix}_mapping/${prefix}_R2.log | samtools view -Shb - > ${prefix}_mapping/${prefix}_R2.bam

hicFindRestSite --fasta $reference_fasta --searchPattern GATC -o $reference_fasta.rest_site_positions.bed

hicBuildMatrix --danglingSequence GATC --samFiles mapped_files/HiC_R1.bam mapped_files/HiC_R2.bam --binSize ${binSize} --restrictionSequence GATC --threads 8 --inputBufferSize 100000 -o matrix/hic_matrix_${binSize}_3.53.h5 --restrictionCutFile ../Ca_59.dna.fa_rest_site_positions.bed --QCfolder QC/${binSize}_3.53
```

Supplementary Method 10. Bisulfite library preparation

About 1ug of genomic DNA spiked with 1 ng unmethylated Lambda DNA was fragmented by sonication to a mean size of approximately 200-500 bp, then end-repaired, 5'-phosphorylated, 3'-dA-tailed, and ligated to 5-methylcytosine-modified adapters. After bisulfite treatment, the DNA was amplified with 10 cycles of PCR using Illumina 8-bp dual index primers. The constructed WGBS libraries were then analyzed by Agilent 2100 Bioanalyzer and finally sequenced on Illumina platforms using a 150×2 paired-end sequencing protocol. Samples were prepared and sequenced with assistance from Shanghai Jiayin Biotechnology Co., Ltd. Methylation level was estimated using Bismark (0.23.1) with the commands:

```
bismark --gzip --parallel 30 --genome . -1 $R1.fq.gz -2 $R2.fq.gz
deduplicate_bismark --bam $R1_bismark_bt2_pe.bam
bismark_methylation_extractor --gzip --bedGraph $R1_bismark_bt2_pe.deduplicated.bam
```

Supplementary Method 11. ChIP assay

Grind young leaf (2g) into a fine powder in liquid nitrogen and then crosslinked with 1% formaldehyde for 10 min at room temperature. After sonication, immunoprecipitation was performed with antibodies. ChIP was performed using antibodies against the following: H3K4me3 (Abcam, ab8580), H3K27me3 (Millipore 07-499), and H3K9me2 (Abcam, ab1220). The immunoprecipitated complex was washed, and DNA was extracted and purified by Universal DNA Purification Kit (QIAquick PCR Purification Kit, 28106). The ChIP-Seq library was prepared using the ChIP-Seq DNA sample preparation kit (NEBNext® Ultra™II DNA) according to the manufacturer's instructions. For ChIP-seq, extracted DNA was ligated to specific adaptors followed by deep sequencing in the Illumina Novaseq 6000 using 150bp paired-end. Samples were prepared and sequenced with assistance from Shanghai Jiayin Biotechnology Co., Ltd.

ChIP-seq mapping and peaks calling were run with the commands:

```
bwa mem -t 24 -M -R “@RG\\tID:${sample}\\tLB:${sample}\\tSM:${sample}\\tPL:ILLUMINA” ${genome_file} ${file1} ${file2} |samtools sort -@ 20 -m 10G > /mnt/memorydisk/${sample}/${sample}.sort.bam

macs2 callpeak -t $prefix.sort.bam -c ${prefix}input.sort.bam -f BAMPE -g 3e9 -n $prefix.contain_input -q 0.05  --shift -100 --extsize 200 --nomodel -B
```

Supplementary Note 1. Analysis of LTR-RTs in the *C. annuum* genome

We initially identified 7,074 full-length LTR-RTs in the CA59 assembly, corresponding to an average of 2.5 elements per megabase (Mb). This density is comparable to those also observed from high-continuous genome assemblies of three other Solanaceous plants, including tomato (3.2 per Mb), eggplant (2.7 per Mb), and potato (4.1 per Mb), but substantially fewer than that in maize, in which we identified as 25 intact LTR-RTs per megabase (Supplementary Fig. 6a). Additionally, although the sequence of LTR-RTs makes up 73.2% of the pepper genome, the identified full-length LTR-RTs only account for 1.9% of the genome (121 Mb). By comparison, the full-length LTR-RT elements (n=51,213) in maize occupy 25% (540 Mb/2.1 Gb) of its genome (Supplementary Fig. 6a). The distribution of estimated insertion times of all full-length LTR-RTs identified from the four Solanaceous species uncovers a very recent surge of LTR-RT amplification in the pepper genome, with 1,234 elements having identical 5' and 3' LTRs (Supplementary Fig. 6b). The intact LTR-RTs in the pepper genome were further grouped into 4,721 families based on alignment above 80% identity and 80% coverage for their long terminal repeat sequences. We found that the majority (75%, 2343/3121) of families have less than five copies, only seven families have copy numbers exceeding 50, of which 6 belong to the *Gypsy* superfamily and 1 belongs to the *Copia* superfamily (Supplementary Fig. 6c). Among the top nine most abundant families, five families have most of their copies with an estimated time of zero (Supplementary Fig. 6d), suggesting these five families account for the recent burst of LTR-RTs in the pepper genome. These observations together suggest a very rapid decay of LTR-RTs occurred in the *C. annuum* genome and the majority of relatively old LTR-RT families have been largely eliminated or fragmented after their periodic amplifications, leaving only a few complete copies in the genome. Further, with a preliminary analysis based on the divergence of pair-wise aligned TE sequences, we identified 4 peaks of LTR divergence in the pepper genome, suggesting the presence of at least 4 bursts of TE activity in the past (Supplementary Fig. 6g).

In an attempt to shed light on the pattern of accumulation and removal of LTR-RTs in the pepper genome, we comprehensively identified LTR-RTs and investigated their structural features. Using a custom annotation pipeline based on homologous and structural characters from the original set of LTR-RT elements, we renewedly identified 10,752 intact elements with flanking target site duplications (TSDs), 6,544 intact elements without TSDs, 8,329 solo-LTRs with TSDs, 35,279 solo-LTRs without TSDs, and 354,257 truncated elements (required to cover at least 80% of the size of the full-length element, or containing at least one LTR) (Supplementary Fig. 6e). These elements totally account for 45% of the genome, leaving a substantial proportion (~34%) of previously annotated LTR-RT sequence

that was not taken into account due to their highly fragmentary structure. Notably, the number of solo-LTRs without TSDs is about four times as those with TSDs suggest that inter-element unequal recombination [21] is more prevalent than intra-element ones in the pepper genome.

Calculating from these newly identified LTR-RT elements, the overall ratio of solo LTRs (S) to intact elements (I) in the pepper genome is 2.52 which is significantly higher than rice[22], soybean[23], maize, and other three Solanaceous species. In contrast to previous reports in tomato and rice, the S/I ratio in recombination-suppressed pericentromeric regions (2.80) is slightly higher than that in gene-rich euchromatic regions (2.29) (Supplementary Fig. 6f). To account for this opposing finding, we separated the LTR-RT families into two categories based on their estimated insertion times. We found that young LTR-RT families (< 1 Mya) are preferentially located in gene-rich euchromatic regions, similar to the previous report in tomato. The S/I ratio calculated only with the young families in recombination-suppressed pericentromeric regions (2.10) is found to be lower than that in gene-rich euchromatic regions (2.69), which is consistent with the assumption that unequal homologous recombination is suppressed in the heterochromatin pericentromeric regions. Surprisingly, the intact elements of the relatively old families are even more enriched in gene-rich euchromatic regions. This result is further confirmed by analyses of individual families. We propose that mechanisms other than unequal recombination processes (URs) have resulted in an even faster decay of LTR-RTs in the pericentromeric regions of the pepper genome and therefore blocked the effect of URs. This assumption was supported by the massive occurrence of partially deleted or truncated elements in the pepper genome. This result is further supported by analyses of both young and old individual families. Taken together, our results suggest that it is illegitimate recombination that predominantly drives the rapid decay of LTR-RTs in the pepper genome, especially in the recombination-suppressed pericentromeric regions.

Supplementary Note 2. Analysis of TAD-like domains inferred by TADtool

We also explored the method TADtool, which is based on the insulation index, to identify TAD-like domains for the pepper genome. We used a leaf Hi-C contact matrix which was corrected by the *BNBC* program for testing and comparing with other methods. Using the optimized parameters (window size: 100 kb and TAD cutoff 2e7), TADtool inferred 2,070 domains, and these domains covered ~75% of the pepper genome (Supplementary Fig. 12a). About ~66% of domains inferred by TADtool can be also found in the other three methods (Supplementary Fig. 12b).

We next applied the TADtool to all eight samples and found that domain calls were largely consistent across tissues both in location and size (as shown in the below figure). A hierarchical clustering analysis based on the conservation of domains and boundaries also demonstrated that domain calls were reproducible across tissues and replicates (Supplementary Fig. 12c,d). Roughly, between 58% and 79% of TAD-like domains, and between 60% and 91% of the boundaries were shared across pairwise sample comparisons (Supplementary Fig. 12c,d). At least 75% of domains identified in one tissue were also detected in other tissues (Supplementary Fig. 12e). Of the domains found only in a single tissue, about 60.2-86.9% are found only in a single replicate whereas 13.0-39.8% (which corresponds to 1.3-4.7% of the total domains) are found in both replicates (Supplementary Fig. 12e). Our results suggest that as much as 1.3-4.7% of TADtool inferred domains might be limited to only one of the tissues investigated here.

Supplementary Note 3. The relationship between gene expression and compartment switching

To explore the relationship between genome organization and gene expression, we assessed whether and to what extent compartment switching corresponds to changes in transcription levels. To do so, we performed a pairwise comparison of both the compartment profiles (8 subcompartments inferred by *Calder*) and the transcriptomes of the four pepper tissues. The finer subcompartments reflect more subtle changes in compartmentalization than the large A and B compartments. In each paired comparison (for which there were six in total), all 40-kb bins (76,641, 3.07 Gb/40 kb, Supplementary Table 16) were assigned into three groups based on the pairwise status of subcompartments: (1) 'down' bins where subcompartment rank decreased by at least 1 in the comparison (e.g. from A1.1 to A1.2 or lower), (2) 'up' bins where subcompartment rank increases by at least 1 (e.g. from B2.2 to B2.1 or higher), and (3) the 'stable' bins in which subcompartment remains unchanged. We also identified between 6,974 and 17,576 differentially expressed genes (DEGs, adjusted *P*-value < 0.01, among 38, 974 testable genes (with CPM > 0.05), Supplementary Table 16) in paired tissue comparisons using the R package Limma [24].

If the subcompartment is related to gene expression, we predict that regions that switch subcompartments would contain more DEGs. Unexpectedly, we observed no enrichment of DEGs in the 'up' or 'down' categories relative to the 'stable' categories (Supplementary Table 16-17). However, the percentage of genes with increased expression did rise from the 'down' to 'up' comparisons and the reverse for genes with decreased expression (Supplementary Table 16-17). This trend was more pronounced when we consider only DEGs (Supplementary Table 16-17). Consistent with this, we

observed that ‘up’ bins overlap genes that exhibited significantly higher log2(fold change) of transcriptional level than those in ‘stable’ bins (Wilcoxon rank-sum test $p < 0.006$ for five comparisons; Fig. 7a), suggesting the ‘up’ bins are associated with increases in gene expression. In contrast, the ‘down’ bins overlapped genes that exhibit significantly lower log2(fold change) value than the ‘stable’ group (Wilcoxon rank-sum test $p < 0.023$ for four comparisons; Fig. 7a), suggesting they are associated with decreases in gene expression. We repeated the analysis using transcription levels measured in bins (i.e. 40 kb) and obtained similar results (Supplementary Table 14-15 and Supplementary Fig. 18a). These results suggest that subcompartment patterning has limited effects on differential gene expression and may instead shape subtle changes in the amplitude of global transcription levels, especially for DEGs.

We also performed the reciprocal analysis to see whether changes in gene expression corresponded to changes in subcompartments. In comparisons between pairs of tissues, all transcribed bins (24,038 testable 40-kb bins with CPM > 0.5) were assigned into three groups based on their changes of transcription level: (1) the down group, in which bins exhibited expression level decreases larger than 2 fold, (2) the up group, in which bins exhibited expression level increases larger than 2 fold, and (3) the stable group that included all other bins. By integrating subcompartment profiles, we observed that bins with increased subcompartment rank were slightly enriched in the up expression group, while bins with decreases in subcompartment rank were slightly enriched in the down group (see Fig. 7b for comparison between bud and leaf, and other five comparisons in Supplementary Fig. 18b). However, a large fraction of bins (e.g. 64.1-65.3% in the comparison between bud and leaf) exhibited stable subcompartment ranks (rank change = 0) in all three groups. These results suggest that changes in gene expression are only associated with subcompartment patterning for a small subset of genomic regions because most differentially transcribed bins remain unchanged subcompartments.

Supplementary Table 1. DNA sequencing data used for *de novo* genome assembly of the *C. annuum* accession CA59.

	PacBio Long reads	MGI-seq short reads	Hi-C[a]
Total reads	22,821,605	1,206,817,158	1,383,854,982
Base num	451,852,814,994 bp (451.9 Gb)	362,045,147,400 bp (362.0 Gb)	415,156,494,600 bp (415.2 Gb)
Depth[b]	~153	~123	~141
Max reads len	366,507 bp	150 bp	150 bp
Mean reads len	19,799 bp	150 bp	150 bp
**Statistic of raw PacBio reads**			
N10	49,949	L10	743,201
N20	41,139	L20	1,749,026
N30	35,729	L30	2,931,408
N40	31,680	L40	4,276,950
N50	**28,351**	L50	5,786,079
N60	25,348	L60	7,471,254
N70	22,103	L70	9,374,916
N80	17,869	L80	11,630,487
N90	12,005	L90	14,666,901
**Statistic of Longest 200Gb PacBio reads**			
Total reads		4,899,539	
Base num		200,000,000,577 bp (200.0 Gb)	
N50	**39,818**	L50	1,987,091

[a]Hi-C data used for genome scaffolding was combined from leaf and bud.

[b]Depth is calculated based on the genome size of 2.95 Gb.

Supplementary Table 2. Summary statistics from the genome assembly processes of the *C. annuum* accession CA59.

**Features**	**Step 1[a]**	**Step 2[b]**	**Step 3[c]**		
	**Contigs**	**Contigs**	**Contigs**		**Scaffolds**
Number	623	623	633		53
Total Bases (bp)	3,077,075,934	3,077,455,690	3,077,455,690		3,077,745,690
N10	153,951,859	153,962,897	153,962,897		333,236,220
N50	41,268,318	41,272,735	41,272,735		262,042,601
N80	8,078,129	8,080,144	8,080,144		250,670,825
N90	3,113,331	3,113,874	3,113,874		178,542,910
Mean length (bp)	4,939,126	4,939,736	4,861,699		58,070,673
Maximum length (bp)	171,532,950	171,547,689	171,547,689		333,236,220
	**Length (bp)**	**Number of contigs**		**Length (bp)**	**Number of contigs**
Chr01	333,203,220	67	Chr07	267,581,468	57
Chr02	178,542,910	29	Chr08	174,069,135	22
Chr03	289,386,686	68	Chr09	280,251,226	32
Chr04	250,670,825	28	Chr10	248,471,258	39
Chr05	254,458,353	42	Chr11	276,018,119	44
Chr06	251,123,559	36	Chr12	262,022,601	`
Unplaced	11,656,330	128			
Quality Value (QV)[d] = -10log10(Probability of error)	52				

[a] Step1: PacBio reads were first corrected by MECAT2, and then trimmed and assembled using CANU. In this step, we obtained an original assembly of 623 gapless contigs.

[b] Step2: The resulting contigs were further polished with short reads three times using Pilon.

[c] Step3: Chromosome conformation capture (Hi-C) data were next used to scaffold the polished contigs using Juicer and 3D-DNA pipeline.

[d] Phred Quality Score was calculated with $QV = -10 \log_{10}P$, where P indicates Probability of error, here P = 17,802 / (3,077,455,690 * 99.455645%). 99.455645% of sites with at least 3 mapped reads. 17,802 valiant sited identified by mapping short reads to the CA59 genome.

Supplementary Table 3. Compassion of assembling quality between our CA59 assembly and five other published genomes of pepper accessions.

**Features**	***C. annuum* (CA59)**	***C. annuum* (Zunla-1)**	***C. annuum* var. *glabriusculum***	***C. annuum* (CM334)**	***C. baccatum***	***C. chinense***
Assembled genome size (bp)	3,077,745,690	3,363,962,270	3,528,040,346	3,063,870,048	3,215,640,822	3,009,382,738
Contigs N50 (bp)	41,272,735	55,436	52,229	29,995	38,843	50,3120
Number of Contigs	633	1,102,811	2,111,345	340,725	257,218	239,428
Maximum size (bp)	171,547,689	705,398	1,246,675	442,125	494,009	872,291
Number of scaffolds	53	13	13	35,801	23,278	50,372
N50 of scaffolds (bp)	262,042,601	229,934,170	229,064,124	250,929,874	229,738,584	237,150,106
Maximum size (bp)	333,236,220	714,758,103	1,074,497,993	309,102,287	297,848,814	275,189,702
Sequence of contigs placed on chromosomes	3,065,925,860	2,649,204,167	2,453,542,353	2,898,262,813	2,818,130,738	2,806,833,320
Percentage of contigs sequence placed on chromosome	99.62%	78.75%	69.54%	94.59%	87.64%	93.27%
BUSCO % of chromosome assembly	95.76%	89.51%	89.38%	87.85%	90.07%	91.39%
Complete and Single-copy BUSCOs	1307	1215	1219	1197	1233	1253
Complete and Duplicated BUSCOs	38	35	32	34	33	33
Fragmented BUSCOs	34	39	36	34	31	30
Missing BUSCOs	61	151	153	175	143	124
Reference	**This study**	[25]	[25]	[26]	[27]	[27]

Supplementary Table 4. Number of intact LTR retrotransposons identified in four *Capsicum* accessions based on EDTA pipeline [18].

Super families	Features	*C. annuum* (CA59)	*C. annuum* (Zunla-1)	*C. annuum* var. *glabriusculum*	*C. annuum* (CM334)
Copia	Number	1,610	1,235	1,056	1,403
	Genome coverage	8.97Mb	6.70Mb	5.78Mb	7.91Mb
Gypsy	Number	3,770	1,470	637	1,294
	Genome coverage	41.5Mb	15.3Mb	5.21Mb	11.9Mb
Unknow	Number	1,694	1,089	1,095	1,460
	Genome coverage	9.44Mb	5.52Mb	5.50Mb	7.71Mb
Total	Number	7,074	3,795	2,789	4,157
	Genome coverage	59.89Mb	27.49Mb	16.5Mb	27.52Mb

Supplementary Table 5. Evidence resource used for gene annotation in the MAKER pipeline.

**Transcription evidence**			
	Number of transcripts	Number of genes	Average length of transcripts (bp)
Merged	76,905	22,477	1,742
leaf	36,143	14,279	1,657
bud	50,237	18,506	1,811
placenta	36,329	13,922	1,481
pulp	36,733	14,085	1,383
root	35,257	12,074	1,943
**Peptide evidence**			
		Number of peptide	
C. annuum (Zunla-1)		35,158	

Supplementary Table 6. Summary of gene annotation (MAKER) and transposable elements (TEs) annotation (EDTA and RepeatMasker) in the current CA59 assembly.

**Gene annotation based on MAKER**			
Number of gene models	46,160	Average length	3,005 bp
**TE annotation based on EDTA and RepeatMasker**			
Super families	Number of elements	Length occupied	Percentage of sequence
LTR	2,643,211	2,253,318,509	73.21%
LTR/Copia	612,279	383,275,322	12.45%
LTR/Gypsy	889,753	1,062,185,071	34.51%
LTR/unknow	1,141,179	807,858,116	26.25%
DNA	1,273,076	353,867,285	11.50%
Total	3,916,287	2,607,185,794	84.71%

Supplementary Table 7. Summary of DNA sequence data generated in this study.

Data type	Tissue	Amount	Purpose	CNGBdb accession
Long-read WGS (PacBio SEQUEL II) (3 SMRT cells)	Young leaf	451.85Gb (~150X of estimated genome coverage)	De novo genome assembly	CNR0255377<br>CNR0255378<br>CNR0255379
Short-read WGS (MGI-seq 2000) (150bp PE)	Young leaf	362.05Gb (~100X of estimated genome coverage)	Genome characteristic survey and contig polishing	CNR0255380
RNAseq (MGI-seq 2000) (150bp PE)	Leaf rep1	6.22Gb	Gene annotation and expression level estimation	CNR0403420
	Leaf rep2	6.42Gb		CNR0403421
	Leaf rep3	8.65Gb		CNR0403422
	Flower bud rep1	7.14Gb		CNR0403411
	Flower bud rep2	6.67Gb		CNR0403412
	Flower bud rep3	6.11Gb		CNR0403413
	Placenta rep1	6.95Gb		CNR0403417
	Placenta rep2	6.99Gb		CNR0403418
	Placenta rep3	6.80Gb		CNR0403419
	Pulp rep1	7.17Gb		CNR0403408
	Pulp rep2	6.18Gb		CNR0403409
	Pulp rep3	7.77Gb		CNR0403410
	Root rep1	7.79Gb		CNR0403414
	Root rep2	6.05Gb		CNR0403415
	Root rep3	6.54Gb		CNR0403416
ISO-seq (PacBio SEQUEL II)	Leaf	554516 ZMWs, 36.23Gb	Gene annotation	CNR0454816
	Flower bud	584010 ZMWs, 38.53Gb		CNR0454817
	Placenta	538919 ZMWs, 34.41Gb		CNR0454818
	Pulp	567153 ZMWs, 36.73Gb		CNR0454819
	Root	515677 ZMWs, 34.32Gb		CNR0454820
Hi-C sequence (MGI-seq 2000) (150bp PE)	Leaf Rep1	208.72Gb	3D genome	CNR0403404
	Leaf Rep2	180.05Gb		CNR0403406
	Flower bud Rep1	206.43Gb		CNR0403401
	Flower bud Rep2	221.51Gb	Hi-C scaffolding (~141x of estimated genome coverage)	CNR0403403
	Placenta Rep1	236.46Gb		CNR0403402
	Placenta Rep2	182.85Gb		CNR0403405
	Pulp Rep1	232.88Gb		CNR0403400
	Pulp Rep2	166.96Gb		CNR0403407
Chip-seq (Novaseq 6000) (150bp PE)	H3K27me3-1input	7.05Gb	Epigenomic analysis	CNR0515149
	H3K27me3-1	6.27Gb		CNR0515150
	H3K27me3-2input	5.22Gb		CNR0515151
	H3K27me3-2	5.93Gb		CNR0515152
	H3K4me3-1input	5.14Gb		CNR0515153
	H3K4me3-1	6.68Gb		CNR0515154
	H3K4me3-2input	7.28Gb		CNR0515155
	H3K4me3-2	7.81Gb		CNR0515156
	H3K9me2-1input	6.78Gb		CNR0515157
	H3K9me2-1	6.42Gb		CNR0515158
	H3K9me2-2input	6.04Gb		CNR0515159
	H3K9me2-2	6.50Gb		CNR0515160
Bisulfite-Seq (Novaseq 6000) (150bp PE)	Bisufic-1	96.70Gb	DNA methylation assay	CNR0515161
	Bisufic-2	91.10Gb		CNR0515162

Supplementary Table 8. Hi-C library statistics with data processed using HiCExplorer.

	**Pulp rep1**	**Pulp rep2**	**Placenta rep1**	**Placenta rep2**	**Bud rep1**	**Bud rep2**	**Leaf rep1**	**Leaf rep2**
**Sequenced read pairs**	776,273,203	556,541,047	788,193,907	609,514,163	738,363,152	688,112,945	695,742,037	600,152,556
**Pairs mappable, unique and high quality**	628,978,882 (81.03%)	449,930,578 (80.84%)	634,788,853 (80.54%)	494,486,532 (81.13%)	563,086,975 (76.26%)	560,055,287 (81.39%)	561,899,621 (80.76%)	483,196,021 (80.51%)
**Valid read pairs (Hi-C Contacts)**	369,107,455 (**47.50%**)	287,509,158 (**51.70%**)	363,207,984 (**46.10%**)	325,669,374 (**53.40%**)	330,648,188 (**44.80%**)	300,026,044 (**43.60%**)	264,494,587 (**38%**)	311,651,182 (**51.90%**)
**Inter-chromosomal**	73,901,222 (9.52%)	103,064,370 (18.52%)	74,132,787 (9.41%)	130,385,093 (21.39%)	112,929,913 (15.29%)	73,219,707 (10.64%)	60,530,352 (8.70%)	139,412,841 (23.23%)
**Intra-chromosomal**	295,206,233 (38.03%)	184,444,788 (33.14%)	289,075,197 (36.68%)	195,284,281 (32.04%)	217,718,275 (29.49%)	226,806,337 (32.96%)	203,964,235 (29.32%)	172,238,341 (28.70%)
**Short range (<20kb)**	24,220,282 (3.12%)	15,423,088 (2.77%)	43,884,349 (5.57%)	15,940,132 (2.62%)	23,837,824 (3.23%)	23,023,999 (3.35%)	21,252,649 (3.05%)	17,511,091 (2.92%)
**Long range (>=20kb)**	270,985,951 (34.91%)	169,021,700 (30.37%)	245,190,848 (31.11%)	179,344,149 (29.42%)	193,880,451 (26.26%)	203,782,338 (29.61%)	182,711,586 (26.26%)	154,727,250 (25.78%)

Supplementary Table 9. Hi-C library statistics with data processed using Juicer.

	Pulp rep1	Pulp rep2	Placenta rep1	Placenta rep2	Bud rep1	Bud rep2	Leaf rep1	Leaf rep2
**Sequenced read pairs**	776,273,203	556,541,047	788,193,907	609,514,163	738,363,152	688,112,945	693,082,972	600,152,556
**Alignable (Normal+Chimeric Paired)**	696,652,687 (89.74%)	517,973,200 (93.07%)	702,032,064 (89.07%)	566,974,526 (93.02%)	594,718,675 (80.55%)	620,807,592 (90.22%)	628,676,341 (90.71%)	556,222,124 (92.68%)
**Valid read pairs (Hi-C Contacts)**	487,387,675 (**62.79%**)	416,617,593 (**74.86%**)	470,923,566 (**59.75%**)	463,887,049 (**76.11%**)	347,146,612 (**47.02%**)	380,975,618 (**55.37%**)	335,109,712 (**48.35%**)	445,908,367 (**74.30%**)
**Inter-chromosomal**	68,262,294 (8.79%)	107,750,067 (19.36%)	64,798,530 (8.22%)	135,652,442 (22.26%)	91,388,379 (12.38%)	68,075,267 (9.89%)	55,641,200 (8.03%)	144,775,810 (24.12%)
**Intra-chromosomal**	419,125,381 (53.99%)	308,867,526 (55.50%)	406,125,036 (51.53%)	328,234,607 (53.85%)	255,758,233 (34.64%)	312,900,351 (45.47%)	279,468,512 (40.32%)	301,132,557 (50.18%)
**Short range (<20kb)**	153,128,050 (19.73%)	134,882,911 (24.24%)	164,715,075 (20.90%)	143,882,640 (23.61%)	81,662,329 (11.06%)	115,264,156 (16.75%)	103,399,508 (14.92%)	142,214,054 (23.70%)
**Long range (>20kb)**	265,996,646 (34.27%)	173,984,368 (31.26%)	241,409,378 (30.63%)	184,351,714 (30.25%)	174,095,727 (23.58%)	197,636,045 (28.72%)	176,068,830 (25.40%)	158,918,159 (26.48%)

Supplementary Table 10. Resolution# of Hi-C contact maps (HiCExplorer) across eight samples.

Samples/Tissues	5 kb	10 kb
Leaf1	875/66,060 (**1.3%**)	19,174/33,222 (**57.7%**)
Leaf2	4,802/66,083 (**7.3%**)	29,162/33,235 (**87.7%)**
Bud1	3,038/66,227 (**4.6%**)	30,499/33,253 (**91.7%**)
Bud2	955/66,089 (**1.4%**)	26,034/33,231 (**78.3%**)
Pulp1	3,150/66,067 (**4.8%**)	29,471/33,217 (**88.7%**)
Pulp2	584/66,054 (**0.9%**)	26,981/33,221 (**81.2%**)
Placenta1	3,506/66,165 (**5.3%**)	28,716/33,251 (**86.4%**)
Placenta2	6,084/66,061 (**9.2%**)	29,064/33,214 (**87.5%**)

#The ‘‘map resolution’’ is defined as the smallest locus size such that 80% of loci have at least 1,000 contacts[28].

According to this, the map resolution of six samples is between 5 kb and 10 kb, and the other two samples are slightly lower than 10-kbresolution.

Supplementary Table 11. QuASAR-QC quality scores for HiC samples.

**#Sample**	**QuASAR-QC value**
Pulp1	0.054
Pulp2	0.051
Bud1	0.048
Bud2	0.057
Placenta1	0.054
Placenta2	0.039
Leaf1	0.061
Leaf2	0.042

Supplementary Table 12. Reproducibility scores of Hi-C data for pairs of samples measured using 3DChromatin_ReplicateQC.

Sample1	Sample2	HiC-Spector	QuASAR-Rep
Pulp1	Pulp2	0.447	0.926
Pulp1	Bud1	0.315	0.786
Pulp1	Bud2	0.307	0.762
Pulp1	Placenta1	0.448	0.865
Pulp1	Placenta2	0.549	0.853
Pulp1	Leaf1	0.269	0.738
Pulp1	Leaf2	0.271	0.689
Pulp2	Bud1	0.224	0.73
Pulp2	Bud2	0.221	0.711
Pulp2	Placenta1	0.283	0.808
Pulp2	Placenta2	0.429	0.86
Pulp2	Leaf1	0.195	0.685
Pulp2	Leaf2	0.205	0.674
Bud1	Bud2	0.818	0.977
Bud1	Placenta1	0.429	0.915
Bud1	Placenta2	0.323	0.855
Bud1	Leaf1	0.491	0.932
Bud1	Leaf2	0.45	0.88
Bud2	Placenta1	0.428	0.915
Bud2	Placenta2	0.321	0.857
Bud2	Leaf1	0.487	0.934
Bud2	Leaf2	0.46	0.905
Placenta1	Placenta2	0.468	0.932
Placenta1	Leaf1	0.351	0.851
Placenta1	Leaf2	0.362	0.835
Placenta2	Leaf1	0.256	0.789
Placenta2	Leaf2	0.286	0.826
Leaf1	Leaf2	0.364	0.841

Supplementary Table 13. Methods used in identifying chromatin loops, corresponding parameters, and the resulting loops.

Methods	Map resolution	Parameters	**Max.** loop distance	**FDR**		Number of the identified loops in tissues (leaf / bud / placenta / pulp)
Mustache						
	10 kb		2 Mb	0.05		1595 / 2063 / 3893 / 510
	15 kb		3 Mb	0.05		1557 / 1696 / 2627 / 393
	20 kb		4 Mb	0.05		1215 / 1079 / 1588 / 255
	25 kb	default	5 Mb	0.05		700 / 527 / 786 / 166
					**Merged:**	**2620 / 2881 / 4790 / 771**
	40 kb		8 Mb	0.05		129 / 102 / 124 / 50
	100 kb		20 Mb	0.05		840 / 847 / 980 / 1057
HiCExplorer / hicDetectLoops						
	10 kb	--windowSize 10 --	8 Mb	0.05		2,832 / 3,241 / 4,115 / 2,418
	15 kb	peakWidth 6	8 Mb	0.05		2,513 / 2,723 / 3,473 / 1,812
	20 kb	--pValuePreselection 0.05	8 Mb	0.05		2,180 / 2,294 / 2,954 / 1,440
	25 kb	--pValue 0.05	8 Mb	0.05		1,904 / 2,033 / 2,477 / 1,192
					**Merged:**	**5990 / 7701 / 9142 / 5746**
	40 kb		20 Mb	0.05		1739 / 1714 / 1737 / 726
	100 kb		20 Mb	0.05		471 / 339 / 307 / 106

Supplementary Table 14. The relationship of subcompartment switching and changes in gene expression between tissues. Expression level was measured per 40-kb bin; *results shown for combined replicates; P values were calculated with one-sided proportion test.*

**Comparisons**	**Num.** of DEBs (q < 0.01)	Number of down bins	Number of stable bins	Number of up bins	*P*-value (Proportion test)
Leaf ***vs.*** Bud	13,099/35,732	12,744(589/1,721) 34.2%[#]<br>(1,139 (66.2%) /582 (33.8%))[&]<br>(491 (83.4%) /98 (16.6%))*	47,917(5,987/15,551) 38.5%<br>(9,171 (59.0%) /6,380 (41.0%))<br>(3,936 (65.7%) /2,051 (34.3%))	15,980(313/930) 33.7%<br>(494 (53.1%) /436 (46.9%))<br>(181 (57.8%) /132 (42.2%))	P=1.00[a] and P=1.00[b]<br>p=4.23e-09[c] | p=0.0002[d]<br>p<2.2e-16[e] | p=0.0025[f]
Leaf ***vs.*** Placenta	11,291/35,732	11,059(334/1,295) 25.8%<br>(454 (35.1%) /841 (64.9%))<br>(198 (59.3%) /136 (40.7%))	41,746(4,686/13,546) 34.6%<br>(5,292 (39.1%) /8,254 (60.9%))<br>(2,382 (50.8%) /2,304 (49.2%))	23,836(967/3,151) 30.7%<br>(934 (29.6%) /2,217 (70.4%))<br>(336 (34.7%) /631 (65.3%))	*P*=1.00 and *P*=1.00<br>*P = 1* | *P < 2.2e-16*<br>*P=0.002* | *P< 2.2e-16*
Leaf ***vs.*** Pulp	10,305/35,732	10,310(325/1,495) 21.7%<br>(1,028 (68.8%) /467 (31.2%))<br>(187 (57.5%) /138 (42.5%))	30,301(2,977/8,832) 33.7%<br>(5,069 (57.4%) /3,763 (42.6%))<br>(1,497 (50.3%) /1,480 (49.7%))	36,230(1,925/7,364) 26.1%<br>(3,976 (54.0%) /3,388 (46.0%))<br>(745 (38.7%) /1,180 (61.3%))	*P*=1.00 and *P*=1.00<br>*p<2.2e-16* | *p=7.64e-6*<br>*p=0.008* | *p=1.18e-15*
Bud ***vs.*** Placenta	16,099/35,732	10,344(407/848) 48.0%<br>(348 (41.0%) /500 (59.0%))<br>(144 (35.4%) /263 (64.6%))	45,745(6,934/15,021) 46.2%<br>(6,102 (40.6%) /8,919 (59.4%))<br>(2,547 (36.7%) /4,387 (63.3%))	20,552(1,706/3,801) 44.9%<br>(1,213 (31.9%) /2,588 (68.1%))<br>(412 (24.2%) /1,294 (75.8%))	*P*=0.16 / *P*=0.92<br>*P=0.42* | *P < 2.2e-16*<br>*P=0.69* | *P < 2.2e-16*
Bud ***vs.*** Pulp	15,694/35,732	9,826(552/1,169) 47.2%<br>(441 (37.7%) /728 (62.3%))<br>(176 (31.9%) /376 (68.1%))	32,086(4,450/9,801) 45.4%<br>(4,148 (42.3%) /5,653 (57.7%))<br>(1,773 (39.8%) /2,677 (60.2%))	32,086(3,791/8,888) 42.7%<br>(2,959 (33.3%) /5,929 (66.7%))<br>(940 (24.8%) /2,851 (75.2%))	*P*=0.13 and *P*=1.00<br>*P =1* | *P < 2.2e-16*<br>*P =1* | *P < 2.2e-16*
Placenta ***vs.*** Pulp	4,787/35,732	11,258(129/1,339) 9.6%<br>(958 (71.5%) /381 (28.5%)<br>(79 (61.2%) /50 (38.3%))	36,369(1,833/11,514) 15.9%<br>(6,979 (60.6%) /4,535 (39.4%))<br>(860 (46.9%) /973 (53.1%))	29,014(578/5,107) 11.3%<br>(3,397 (66.5%) /1,710 (33.5%))<br>(256 (44.3%) /322 (55.7%))	*P*=1.00 and *P*=1.00<br>P= 4.2e-15 | P =1<br>p= 0.0011 | p= 0. 145

[a]prop.test(x = c(589, 5987), n = c(1721, 15551), alternative = c("greater"), conf.level = 0.95, correct = TRUE)

[b]prop.test(x = c(313, 5987), n = c(930,15551), alternative = c("greater"), conf.level = 0.95, correct = TRUE)

[c]prop.test(x = c(1139, 9171), n = c(1721, 15551), alternative = c("greater"), conf.level = 0.95, correct = TRUE)

[d]prop.test(x = c(436, 6380), n = c(930, 15551), alternative = c("greater"), conf.level = 0.95, correct = TRUE)

[e]prop.test(x = c(491, 3936), n = c(589, 5987), alternative = c("greater"), conf.level = 0.95, correct = TRUE)

[f]prop.test(x = c(132, 2051), n = c(313, 5987), alternative = c("greater"), conf.level = 0.95, correct = TRUE)

[#]A total of 12,744 bins with subcompartment switching from higher ranks to low ranks were supported by two replicates. They overlapped with 1,721 testable bins, of them 589 bins are differentially expressed between the two compared tissues.

[&]Among the 1,721 testable bins, 1,139 bins show decreased expression in the first tissue relative to the second, and 582 bins with increased expression in the first tissue relative to the second.

[*]Among the 589 differentially transcribed bins, 491 bins show decreased expression in the first tissue relative to the second, and 98 bins with increased expression in the first tissue relative to the second.

Supplementary Table 15. The relationship of subcompartment switching and changes in gene expression between tissues. Expression level was measured per 40-kb bin; *results shown for a single replicate; P values were calculated with one-sided proportion test.*

**Comparisons**	**Num.** of DEBs (q < 0.01)	Number of down bins	Number of stable bins	Number of up bins	*P*-value (Proportion test)
Leaf ***vs.*** Bud	13,099/35,732	12,744(1,930/5,441) 35.5%<br>(3,559 (65.4%) /1,882 (34.6%))<br>(1,527 (79.1%) /403 (20.9%))	47,917(8,439/22,721) 37.1%<br>(13,693 (60.3%) /9,028 (39.7%))<br>(5,765 (68.3%) /2,674 (31.7%))	15,980(2,665/7,341) 36.3%<br>(4,143 (56.4%) /3,198 (43.6%))<br>(1,693 (63.5%) /972 (36.5%))	*P*=0.99 and *P*=0.90<br>P=1.46e-12 and P=3.45e-9<br>P<2.20e-16 and P=2.52e-6
Leaf ***vs.*** Placenta	11,291/35,732	11,059(1,472/4,892) 30.1%<br>(1,762 (36.0%) /3,130 (64.0%))<br>(787 (53.5%) /685 (46.5%))	41,746(6,434/19,926) 32.3%<br>(7,464 (37.5%) /12,462 (62.5%))<br>(3,210 (49.9%) /3,224 (50.1%))	23,836(3,301/10,685) 30.9%<br>(3,340 (31.1%) /7,345 (68.7%))<br>(1,293 (39.2%) /2,008 (60.8%))	*P*=1.00 and *P*=0.99<br>*P=0.97 and P<2.20e-16*<br>*P=7.25e-3 and P<2.20e-16*
Leaf ***vs.*** Pulp	10,305/35,732	10,310(1,024/3,793) 27.0%<br>(2,399 (63.2%) /1,394 (36.8%))<br>(546 (53.3%) /478 (46.7%))	30,301(4,287/13,962) 30.7%<br>(8,180 (58.6%) /5,782 (41.4%))<br>(2,150 (50.2%) /2,137 (49.8%))	36,230(4,825/17,748) 27.2%<br>(9,702 (54.7%) /8,046 (45.3%))<br>(1,990 (41.2%) /2,835 (58.8%))	*P*=1.00 and *P*=1.00<br>*P=1.18e-7 and 1.47e-12*<br>*P=3.69e-2 and P<2.20e-16*
Bud ***vs.*** Placenta	16,099/35,732	10,344(2,120/4,707) 45.0%<br>(1,824 (38.8%) /2,883 (61.2%))<br>(721 (34.0%) /1399 (66.0%))	45,745(9,935/21,857) 45.5%<br>(8,500 (38.9%) /13,357 (61.1%))<br>(3,375 (34.0%) /6,560 (66.0%))	20,552(3,898/8,939) 43.6%<br>(2,990 (33.4%) /5,949 (66.6%))<br>(951 (24.4%) /2,947 (75.6%))	*P*=0.69 and *P*=1.00<br>P=0.56 and P<2.20e-16<br>*P=0.497 and P<2.20e-16*
Bud ***vs.*** Pulp	15,694/35,732	9,826(1,680/3,630) 46.3%<br>(1,476 (40.7%) /2,154 (59.3%))<br>(609 (36.3%) /1071 (63.8%))	32,086(6,725/15,059) 44.7%<br>(6,144 (40.8%) /8,915 (59.2%))<br>(2,487 (37.0%) /4,238 (63.0%))	34,729(7,127/16,814) 42.4%<br>(5,830 (34.7%) /10,984 (65.3%))<br>(1,874 (26.3%) /5,253 (73.7%))	*P*=0.04 and *P*=1.00<br>*P=0.55 and P<2.20e-16*<br>*P=0.70 and P<2.20e-16*
Placenta ***vs.*** Pulp	4,787/35,732	11,258(409/3,866) 10.6%<br>(2,739 (70.8%) /1,127 (29.2%))<br>(229 (56.0%) /180 (44.0%))	36,369(2,516/17,269) 14.6%<br>(10,805 (62.6%) /6,464 (37.4%))<br>(1,209 (48.1%) /1,307 (51.9%))	29,014(1,678/14,368) 11.7%<br>(9,345 (65.0%) /5,023 (35.0%))<br>(754 (44.9%) /924 (55.1%))	*P*=1.00 and *P*=1.00<br>P<2.20e-16 and P=1<br>P=1.72e-3 and P=2.55e-2

Supplementary Table 16. The relationship of subcompartment switching and changes in gene expression between tissues. Expression level was measured per gene; *results shown for a single replicate; P values were calculated with one-sided proportion test.*

**Comparisons**	**Num.** of DEGs (*q* < 0.01)	Number of down bins	Number of stable bins	Number of up bins	*P*-value (**Two-proportions z-test**)
Leaf ***vs.*** Bud	14,092/38,974	12,744(1,888/5,331)35.4%<br>(3,052(57.3%)/2,279(42.7%))<br>(1,294(68.5%)/594(31.5%))	47,917(9,599/26,295)36.5%<br>(14,379(54.7%)/11,916(45.3%))<br>(5,922(61.7%)/3,677(38.3%))	15,980(2,713/7,644)35.5%<br>(4,031(52.7%)/3,613(47.3%))<br>(1,571(57.9%)/1,142(42.1%))	*P=0.93[a] and P=0.95[b]*<br>*P=3.13e-4[c] and P=1.35e-3[d]*<br>*P=1.08e-08[e] and P=1.95e-4[f]*
Leaf ***vs.*** Placenta	15,391/38,974	11,059(2,179/5,709)38.2%<br>(2,271(39.8%)/3,438(60.2%))<br>(1,054(48.4%)/1,125(51.6%))	41,746(9,629/23,948)40.2%<br>(9,735(40.7%)/14,213(59.3%))<br>(4,543(47.2%)/5,086(52.8%))	23,836(3,692/9,584)38.5%<br>(3,327(34.7%)/6,257(65.3%))<br>(1,465(39.7%)/2,227(60.3%))	*P=1.00 and P=1.00*<br>*P=0.88 and P<2.20e-16*<br>*P=0.16 and P=4.02e-15*
Leaf ***vs.*** Pulp	14,183/38,974	10,310(1,666/4,550)36.6%<br>(2,480(54.5%)/2,070(45.5%))<br>(802(48.1%)/864(51.9%))	30,301(6,896/18,118)38.1%<br>(9,507(52.5%)/8,611(47.5%))<br>(3,092(44.8%)/3,804(55.2%))	36,230(5,728/16,556)34.6%<br>(8,500(51.3%)/8,056(48.7%))<br>(2,329(40.7%)/3,399(59.3%))	*P=0.96 and P=1*<br>*P=7.36e-3 and P=1.8e-2*<br>*P=8.16e-3 and P=1.28e-6*
Bud ***vs.*** Placenta	17,423/38,974	10,344(2,559/5,742)44.6%<br>(2,491(43.4%)/3,251(56.6%))<br>(984(38.5%)/1,575(61.5%))	45,745(11,628/25,964)44.8%<br>(11,090(42.7%)/14,874(57.3%))<br>(4,287(36.9%)/7,341(63.1%))	20,552(3,381/7,572)44.7%<br>(2,891(38.2%)/4,681(61.8%))<br>(999(29.5%)/2,382(70.5%))	*P=0.61 and P=0.58*<br>*P=0.18 and P=1.06e-12*<br>*P=6.96e-2 and P=2.57e-15*
Bud ***vs.*** Pulp	17,576/38,974	9,826(2,146/4,606)46.6%<br>(2,022(43.9%)/2,584(56.1%))<br>(823(38.4%)/1,323(61.6%))	32,086(8,876/19,295)46.0%<br>(8,296(43.0%)/10,999(57.0%))<br>(3,377(38.0%)/5,499(62.0%))	34,729(6,690/15,347)43.6%<br>(6,097(39.7%)/9,250(60.3%))<br>(2,115(31.6%)/4,575(68.4%))	*P=0.24 and P=1*<br>*P=0.14 and P=4.68e-10*<br>*P=0.41 and P<2.20e-16*
Placenta ***vs.*** Pulp	6,974/38,974	11,258(581/3,490)16.6%<br>(2,178(62.4%)/1,312(37.6%))<br>(294(50.6%)/287(49.4%))	36,369(4,267/22,134)19.3%<br>(12,843(58.0%)/9,291(42.0%))<br>(2,018(47.3%)/2,249(52.7%))	29,014(2,170/13,646)15.9%<br>(8,421(61.7%)/5,225(38.3%))<br>(991(45.7%)/1,179(54.3%))	*P=1.00 and P=1*<br>*P=5.64e-07 and P=1*<br>*P=0.07 and P=0.11*

Supplementary Table 17. The relationship of subcompartment switching and changes in gene expression between tissues. Expression level was measured per gene; *results shown for combined replicates; P values were calculated with one-sided proportion test.*

**Comparisons**	**Num.** of DEGs<br>(q < 0.01)	Number of down bins	Number of stable bins	Number of up bins	*P*-value<br>(Proportion test)
Leaf ***vs.*** Bud	14,092/38,974	4,460 (471/ 1,331) 35.4%<br>(785 (59.0%) / 546 (41.0%))<br>(338 (71.8%)/ 133 (28.2%))	31,664 (7,252/ 19,425) 37.3%<br>(10,507(54.1%)/ 8,918(45.9%))<br>(4,364(60.2%)/ 2,888 (39.8%))	1,901 (358/ 1,171) 30.6%<br>(591(50.5%)/581(49.6%))<br>(186(52.0%)/172(48.0%))	*P*=0.92 and *P*=1.00<br>*P*=2.96e-4 and P=7.34e-3<br>*P*=3.83e-7 and P=1.18e-3
Leaf ***vs.*** Placenta	15,391/38,974	3,670 (468/1,202) 38.9%<br>(514 (42.8%)/ 688 (57.2%))<br>(253 (54.1%)/ 215 (45.9%))	27,199 (7,364/17,672) 41.7%<br>(7,365(41.7%)/10,307(58.3%))<br>(3,511(47.7%)/3,853(52.3%))	6,558 (1,045/2,781) 37.6%<br>(906(32.6%)/ 1,875(67.4%))<br>(368(35.2%)/ 677(64.8%))	*P*=0.97 and *P*=1.00<br>*P=0.239 and P<2.20e-16*<br>*P=4.25e-3 and P=2.54e-14*
Leaf ***vs.*** Pulp	14,183/38,974	4,926 (451/1,313) 34.3%<br>(754 (57.4%)/ 559 (42.6%))<br>(233 (51.7%)/ 218 (48.3%))	17,624 (5,149/13,108) 39.3%<br>(6,734(51.4%)/ 6,374(48.6%))<br>(2,284(44.4%)/ 2,865(55.6%))	14,387 (2,308/6,948) 33.2%<br>(3,564(51.3%)/3,384(48.7%))<br>(916(39.7%)/1,392(60.3%))	*P*=1.00 and *P*=1.00<br>*P=1.62e-5 and P=0.464*<br>*P=1.64e-3 and P=9.15e-5*
Bud ***vs.*** Placenta	17,423/38,974	2,164 (471/1,075) 43.8%<br>(468 (43.5%)/ 607 (56.5%))<br>(187 (39.7%)/ 284 (60.3%))	30,567 (8,723/19,471) 44.8%<br>(8,462(43.5%)/11,009(56.5%))<br>(3,327(38.1%)/5,396(61.9%))	8,047 (1,427/3,193) 44.7%<br>(1,184(37.1%)/2,009(62.9%))<br>(420(29.4%)/1,007(70.6%))	*P*=0.73 and *P*=0.54<br>*P=0.49 and P=8.07e-12*<br>*P=0.264 and P=1.59e-10*
Bud ***vs.*** Pulp	17,576/38,974	3,594(609/1,323) 46.0%<br>(573 (43.3%)/ 750 (56.7%))<br>(229 (37.6%)/ 380 (62.4%))	19,949 (6,470/14,028) 46.1%<br>(6,104(43.5%)/7,924(56.5%))<br>(2,523(39.0%)/3,947(61.0%))	17,652 (3,510/8,076) 43.5%<br>(3,172(39.3%)/4,904(60.7%))<br>(1,089(31.0%)2,421(69.0%))	*P*=0.51 and *P*=1.00<br>*P=0.545 and P=4.36e-10*<br>*P=0.736 and P=1.52e-15*
Placenta ***vs.*** Pulp	6,974/38,974	4,049 (182/ 1,114) 16.3%<br>(713 (64.0%)/ 401 (36.0%))<br>(104 (57.1%)/ 78 (42.9%))	22,912 (3197/16,211) 19.7%<br>(9,223(56.9%)/6,988(43.1%))<br>(1,478(46.2%)/1,719(53.8%))	10,462 (718/4,767) 15.1%<br>(3,015(63.2%)/1,752(36.8%))<br>(328(45.7%)/390(54.3%))	*P*=1.00 and *P*=1.00<br>P=2.01e-6 and P=1.00<br>P=2.61e-3 and P=0.41

Supplementary Table 18. Remodeling TAD-like domains and their boundaries is not associated with differential gene expression between pepper tissues.

**Comparisons**	**Num.** of DEGs (q < 0.01)	Conserved TADs/Boundaries	Remodeled TADs/Boundaries	P-value (Proportion test)
Leaf ***vs.*** Bud	14,092/38,974[a]	1,835 | 1,155[b]	1,562 | 3 ,050[c]	
		9,452 / 26,210[d]	4,564 / 12,584[e]	P = 0.7013[f]
		450 / 1,370[g]	990 / 2,777[h]	P = 0.08033
Leaf ***vs.*** Placenta	15,391/38,974	1,623 | 922	2,066 | 3,576	
		9,481 / 24,073	5,974 / 15,072	P=0.6271
		522 / 1,171	1,323 / 3,075	P=0.3801
Leaf ***vs.*** Pulp	14,183/38,974	1,385 | 672	2,276 | 3,801	
		7,634 / 21,258	6,684 / 18,022	P=0.01621
		344 / 907	1,368 / 3,447	P=0.3539
Bud ***vs.*** Placenta	17,423/38,974	1,831 | 1,241	1,602 | 2,892	
		11,799 / 26,399	5,573 / 12,441	P=0.861
		705 / 1,495	1,130 / 2,571	P=0.05144
Bud ***vs.*** Pulp	17,576/38,974	1,604 | 987	1,790 | 3,125	
		10,519 / 23523	7,032 / 15,435	P=0.105
		519 / 1,189	1,301 / 3,001	P=0.888
Placenta ***vs.*** Pulp	6,974/38,974	1,858 | 1,429	1,362 | 2,301	
		4,977 / 28,202	1,919 / 10,509	P=0.1656
		314 / 1,640	362 / 2,036	P=0.3076

[a]Of those 38,974 testable genes, 14,092 genes are differentially expressed between leaf and bud.

[b]Between leaf and bud, there are 1,835 TAD-like folding domains and 1,155 domain boundaries identified are shared; and [c]1,562 domains and 3,050 boundaries identified are specific to either tissue, which we term remodeled TAD features. Notably, here we used a more stringent cutoff to define the conservation of boundaries that is boundaries should be completely overlapped.

[d]There are 26, 210 genes located within the conserved TAD-like folding domains, of them, 9,452 genes are differentially expressed (adjusted P-value < 0.01); and [e] there are 12,584 genes located within remodeled TAD-like folding domains, of them, 4,564 gare differentially expressed.

[g]There are 1,370 genes overlap with TAD-like folding domain boundaries, of them, 450 genes are differentially expressed (adjusted P-value < 0.01);

and [h] there are 2,777 genes overlap remodeled boundaries, of them, 990 are differentially expressed.

[f]p-value was calculated by two-sided proportion test:
prop.test(x=c(9452,4564),n=c(26210,12584),conf.level=0.95,correct=TRUE)

Supplementary Table 19. The relationship of chromatin loops and gene expression between tissues.

**Comparisons**	**Num.** of DEGs (*q* < 0.01)	**Num.** of loops shared at least in two tissues (5,728)	**Num.** of loops specific to a single tissue (13,793)	*P*-value (Proportion test)
Leaf *vs.* Bud	14,092/38,974	2,419/7,048	2,929/8,431	*P* = 0.5968[a]
Leaf *vs.* Placenta	15,391/38,974	2,965/7,048	3,421/8,431	*P* = 0.06265
Leaf *vs.* Pulp	14,183/38,974	2,708/7,048	3,185/8,431	*P* = 0.42
Bud *vs.* Placenta	17,423/38,974	3,145/7,048	3,696/8,431	*P* = 0.3359
Bud *vs.* Pulp	17,576/38,974	3,095/7,048	3,769/8,431	*P* = 0.332
Placenta *vs.* Pulp	6,974/38,974	1273/7,048	1,481/8,431	*P* = 0.4342

[a]p-value was calculated by two-sided proportion test.

prop.test(x=c(2419,2929),n=c(7048,8431),conf.level=0.95,correct=TRUE)

Supplementary Table 20. Sample information for Hi-C and RNA-seq experiments.

Samples No.	Plant tissues	Sample timing	Description
Leaf1*	Young leaves	2020.4.29	Young leaves mixed from 5 plants
Placenta1*	Placentas	2020.4.29	Fruits of 21day after flower mixed from 5 plants
Pulp1*	Pulp	2020.4.29	Fruits of 21day after flower mixed from 5 plants
Bud1*	Flower buds	2020.4.29	Big flower buds mixed from 5 plants
Leaf2	Young leaves	2021.1.13	Young leaves mixed from 5 plants
Placenta2	Placentas	2021.1.13	Fruits of 21day after flower mixed from 5 plants
Pulp2	Pulp	2021.1.13	Fruits of 21day after flower mixed from 5 plants
Bud2#	Flower buds	2020.4.29	Big flower buds mixed from 5 plants

* These samples were used for RNA-seq.

# Bud2 and Bud1 were collected at the same time and therefore were treated as samples in the same batch.

Supplementary Table 21. Public Hi-C data used for Supplementary Fig. 1.

Species	Genome assembly	NCBI accessions	Data source reference
Rice (*Oryza sativa*)	Nipponbare	https://www.ncbi.nlm.nih.gov/search/all/?term=SRR5046931<br>https://www.ncbi.nlm.nih.gov/search/all/?term=SRR5046932	29
Tomato (*Solanum lycopersicum*)	SL4	https://www.ncbi.nlm.nih.gov/search/all/?term=SRR5748725<br>https://www.ncbi.nlm.nih.gov/search/all/?term=SRR5748726<br>https://www.ncbi.nlm.nih.gov/search/all/?term=SRR5748729<br>https://www.ncbi.nlm.nih.gov/search/all/?term=SRR5748730<br>https://www.ncbi.nlm.nih.gov/search/all/?term=SRR5748731<br>https://www.ncbi.nlm.nih.gov/search/all/?term=SRR5748732<br>https://www.ncbi.nlm.nih.gov/search/all/?term=SRR5748733<br>https://www.ncbi.nlm.nih.gov/search/all/?term=SRR5748734<br>https://www.ncbi.nlm.nih.gov/search/all/?term=SRR5748735<br>https://www.ncbi.nlm.nih.gov/search/all/?term=SRR5748736	30
Maize (*Zea mays*)	B73	https://www.ncbi.nlm.nih.gov/search/all/?term=SRR5748747<br>https://www.ncbi.nlm.nih.gov/search/all/?term=SRR5748748<br>https://www.ncbi.nlm.nih.gov/search/all/?term=SRR5748749<br>https://www.ncbi.nlm.nih.gov/search/all/?term=SRR5748750<br>https://www.ncbi.nlm.nih.gov/search/all/?term=SRR5748751<br>https://www.ncbi.nlm.nih.gov/search/all/?term=SRR5748752<br>https://www.ncbi.nlm.nih.gov/search/all/?term=SRR5748753<br>https://www.ncbi.nlm.nih.gov/search/all/?term=SRR5748754<br>https://www.ncbi.nlm.nih.gov/search/all/?term=SRR5748755<br>https://www.ncbi.nlm.nih.gov/search/all/?term=SRR5748756<br>https://www.ncbi.nlm.nih.gov/search/all/?term=SRR5748767<br>https://www.ncbi.nlm.nih.gov/search/all/?term=SRR5748768<br>https://www.ncbi.nlm.nih.gov/search/all/?term=SRR5748769<br>https://www.ncbi.nlm.nih.gov/search/all/?term=SRR5748770<br>https://www.ncbi.nlm.nih.gov/search/all/?term=SRR5748771<br>https://www.ncbi.nlm.nih.gov/search/all/?term=SRR5748772<br>https://www.ncbi.nlm.nih.gov/search/all/?term=SRR5748773	30
Fruit fly (*Drosophila melanogaster*)	dm6	https://www.ncbi.nlm.nih.gov/search/all/?term=GSM3475692<br>https://www.ncbi.nlm.nih.gov/search/all/?term=GSM3475693	31
Human (Homo sapiens)	hg38	https://www.ncbi.nlm.nih.gov/search/all/?term=SRR1030718<br>https://www.ncbi.nlm.nih.gov/search/all/?term=SRR1030719<br>https://www.ncbi.nlm.nih.gov/search/all/?term=SRR1030720	32

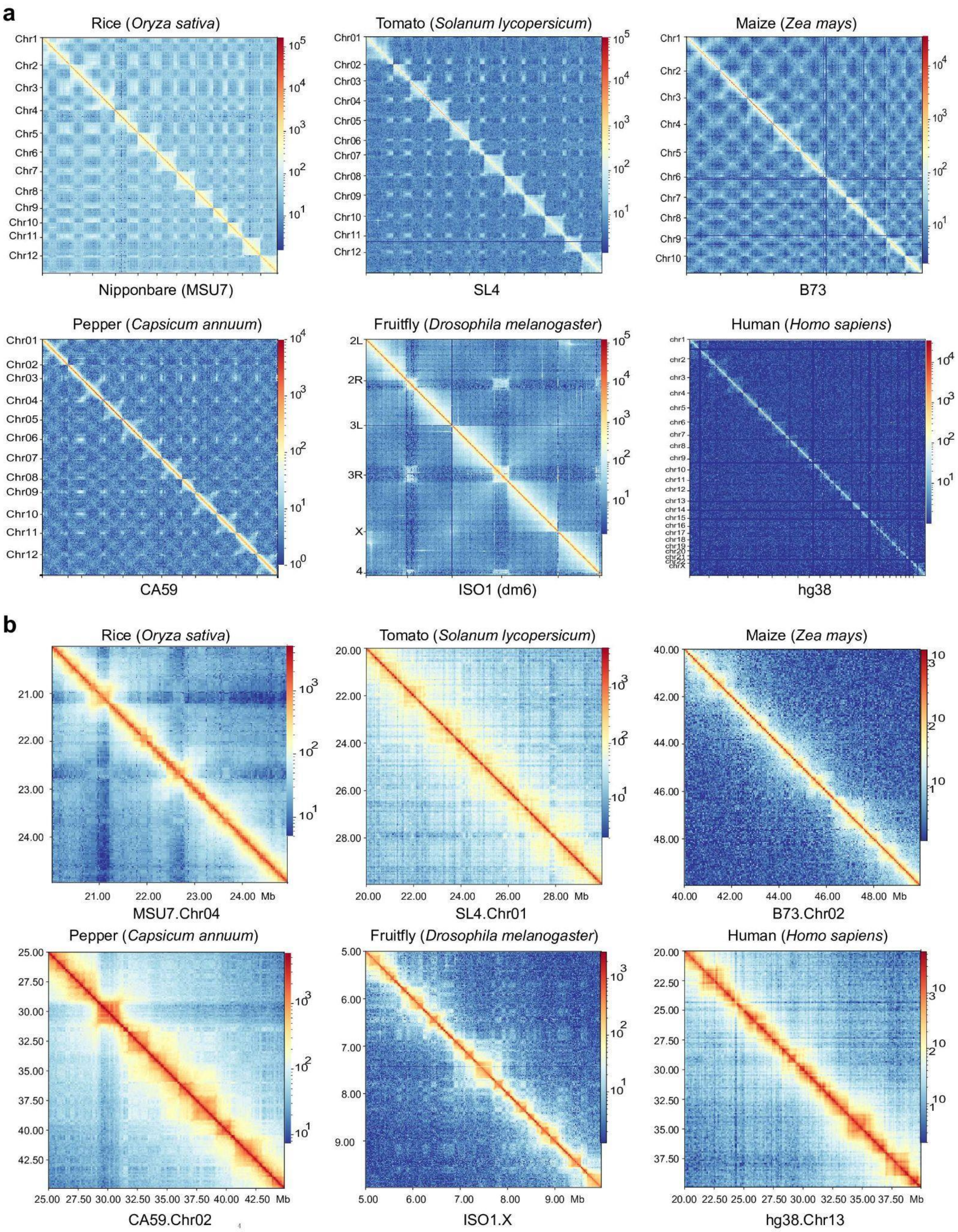

a
Rice (Oryza sativa)
Tomato (Solanum lycopersicum)
Maize (Zea mays)
Nipponbare (MSU7)
SL4
B73
Pepper (Capsicum annuum)
Fruitfly (Drosophila melanogaster)
Human (Homo sapiens)
CA59
ISO1 (dm6)
hg38
b
Rice (Oryza sativa)
Tomato (Solanum lycopersicum)
Maize (Zea mays)
MSU7.Chr04
SL4.Chr01
B73.Chr02
Pepper (Capsicum annuum)
Fruitfly (Drosophila melanogaster)
Human (Homo sapiens)
CA59.Chr02
ISO1.X
hg38.Chr13

Supplementary Fig. 1. A preliminary visual inspection of Hi-C heatmaps in rice, tomato, maize, pepper, fruit fly, and human. **a,** Genome-wide Hi-C heatmaps. Hi-C map resolution for each species: rice, 100 kb; tomato, 100 kb; maize, 500 kb; pepper, 500 kb; *Drosophila*, 100 kb; human 100 kb. **b,** TADs or similar structures (i.e. appear as clearly visible squares in the Hi-C maps) are shown on example regions for each species using higher resolution Hi-C maps. Resolution: rice, 10 kb; tomato, 40 kb; maize, 100 kb; pepper, 100 kb; *Drosophila*, 5 kb; human 40 kb. Published Hi-C data used to generate the Hi-C maps can be found in Supplementary Table 21.

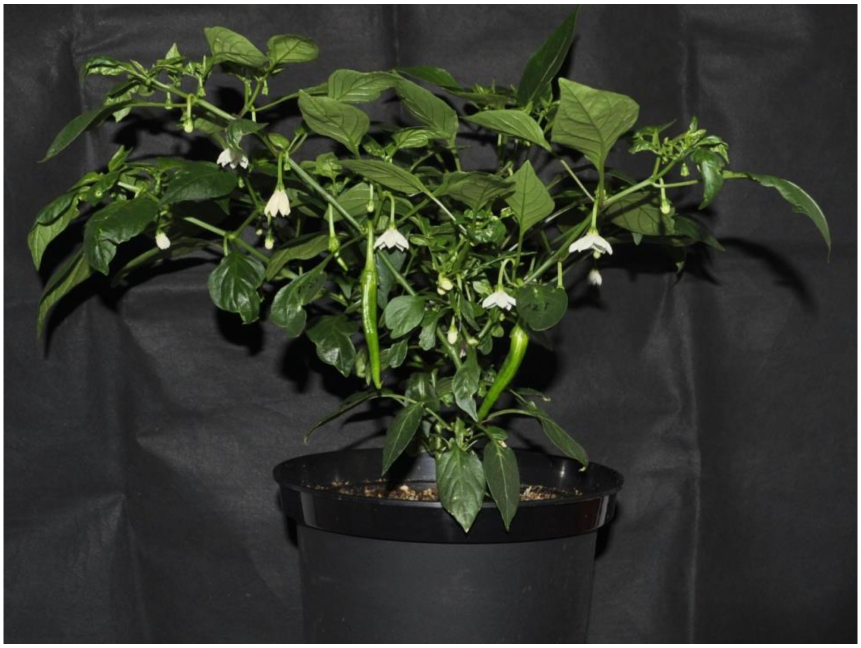

Supplementary Fig. 2. An image of the CA59 accession plant.

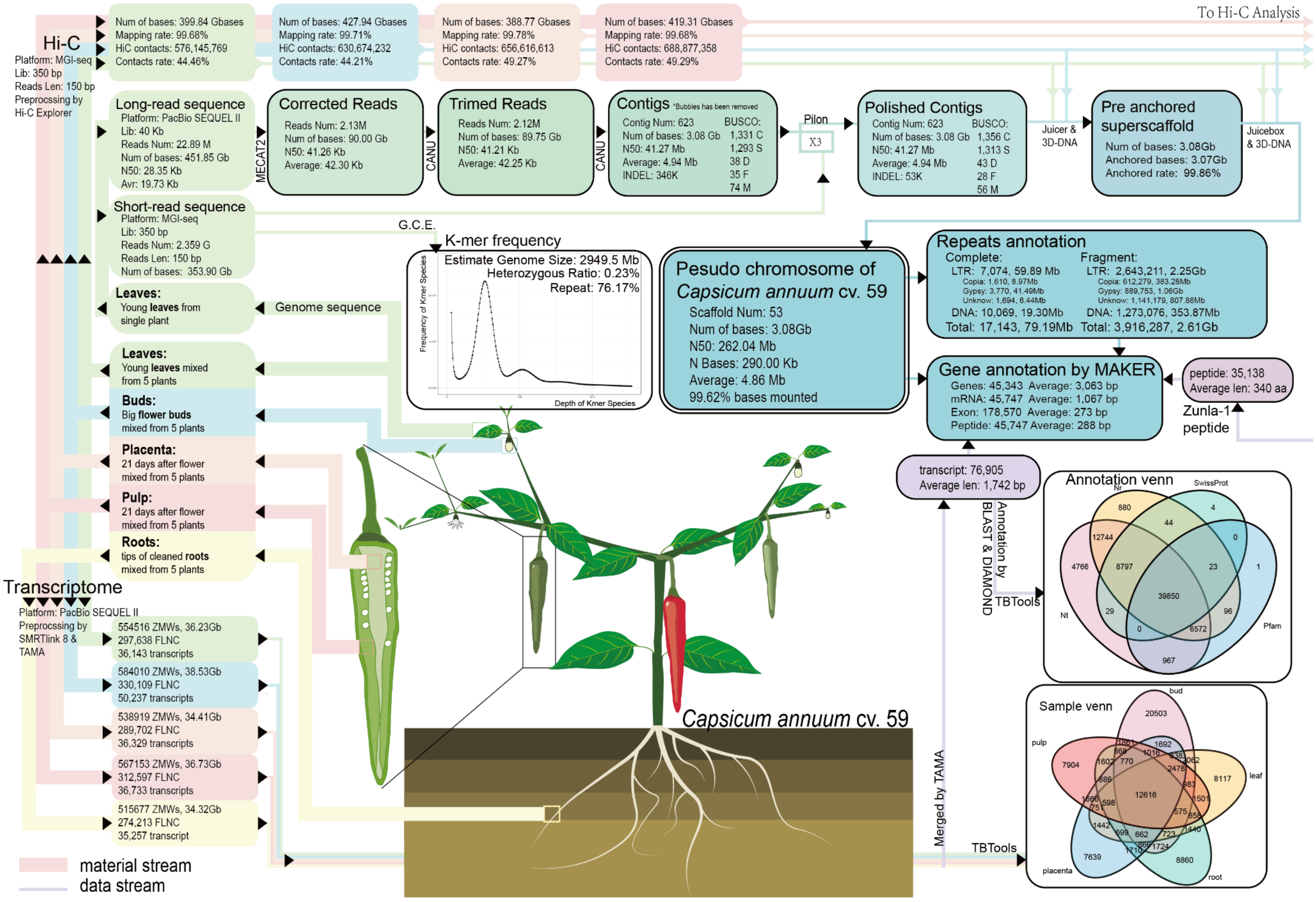

To Hi-C Analysis
Hi-C
Platform: MGI-seq
Lib: 350 bp
Reads Len: 150 bp
Preprocssing by Hi-C Explorer
Num of bases: 399.84 Gbases
Mapping rate: 99.68%
HiC contacts: 576,145,769
Contacts rate: 44.46%
Num of bases: 427.94 Gbases
Mapping rate: 99.71%
HiC contacts: 630,674,232
Contacts rate: 44.21%
Num of bases: 388.77 Gbases
Mapping rate: 99.78%
HiC contacts: 656,616,613
Contacts rate: 49.27%
Num of bases: 419.31 Gbases
Mapping rate: 99.68%
HiC contacts: 688,877,358
Contacts rate: 49.29%
Long-read sequence
Platform: PacBio SEQUEL II
Lib: 40 Kb
Reads Num: 22.89 M
Num of bases: 451.85 Gb
N50: 28.35 Kb
Avr: 19.73 Kb
MECAT2
Corrected Reads
Reads Num: 2.13M
Num of bases: 90.00 Gb
N50: 41.26 Kb
Average: 42.30 Kb
CANU
Trimed Reads
Reads Num: 2.12M
Num of bases: 89.75 Gb
N50: 41.21 Kb
Average: 42.25 Kb
CANU
Contigs *Bubbles has been removed
Contig Num: 623
Num of bases: 3.08 Gb
N50: 41.27 Mb
Average: 4.94 Mb
INDEL: 346K
BUSCO:
1,331 C
1,293 S
38 D
35 F
74 M
Pilon
X3
Polished Contigs
Contig Num: 623
Num of bases: 3.08 Gb
N50: 41.27 Mb
Average: 4.94 Mb
INDEL: 53K
BUSCO:
1,356 C
1,313 S
43 D
28 F
56 M
Juicer & 3D-DNA
Pre anchored superscaffold
Num of bases: 3.08Gb
Anchored bases: 3.07Gb
Anchored rate: 99.86%
Juicebox & 3D-DNA
Short-read sequence
Platform: MGI-seq
Lib: 350 bp
Reads Num: 2.359 G
Reads Len: 150 bp
Num of bases: 353.90 Gb
G.C.E.
K-mer frequency
Estimate Genome Size: 2949.5 Mb
Heterozygous Ratio: 0.23%
Repeat: 76.17%
Frequency of Kmer Species
Depth of Kmer Species
Leaves:
Young leaves from single plant
Genome sequence
Pesudo chromosome of
Capsicum annuum cv. 59
Scaffold Num: 53
Num of bases: 3.08Gb
N50: 262.04 Mb
N Bases: 290.00 Kb
Average: 4.86 Mb
99.62% bases mounted
Repeats annotation
Complete:
LTR: 7,074, 59.89 Mb
Copia: 1,610, 8.97Mb
Gypsy: 3,770, 41.49Mb
Unknow: 1,694, 6.44Mb
DNA: 10,069, 19.30Mb
Total: 17,143, 79.19Mb
Fragment:
LTR: 2,643,211, 2.25Gb
Copia: 612,279, 383.28Mb
Gypsy: 889,753, 1.06Gb
Unknow: 1,141,179, 807.86Mb
DNA: 1,273,076, 353.87Mb
Total: 3,916,287, 2.61Gb
Gene annotation by MAKER
Genes: 45,343 Average: 3,063 bp
mRNA: 45,747 Average: 1,067 bp
Exon: 178,570 Average: 273 bp
Peptide: 45,747 Average: 288 bp
peptide: 35,138
Average len: 340 aa
Zunla-1 peptide
Leaves:
Young leaves mixed from 5 plants
Buds:
Big flower buds mixed from 5 plants
Placenta:
21 days after flower mixed from 5 plants
Pulp:
21 days after flower mixed from 5 plants
Roots:
tips of cleaned roots mixed from 5 plants
transcript: 76,905
Average len: 1,742 bp
Annotation by BLAST & DIAMOND
TBTools
Annotation venn
Nr
SwissProt
Nt
Pfam
Transcriptome
Platform: PacBio SEQUEL II
Preprocssing by SMRTlink 8 & TAMA
554516 ZMWs, 36.23Gb
297,638 FLNC
36,143 transcripts
584010 ZMWs, 38.53Gb
330,109 FLNC
50,237 transcripts
538919 ZMWs, 34.41Gb
289,702 FLNC
36,329 transcripts
567153 ZMWs, 36.73Gb
312,597 FLNC
36,733 transcripts
515677 ZMWs, 34.32Gb
274,213 FLNC
35,257 transcript
Capsicum annuum cv. 59
Merged by TAMA
TBTools
Sample venn
bud
pulp
leaf
placenta
root
material stream
data stream

Supplementary Fig. 3. *De novo* sequencing, assembling, and annotation of the CA59 genome based on PacBio long reads and chromosome conformation capture (Hi-C). (1) For genome sequencing, we collected 451.85 Gb PacBio long reads, 353.9 Gb short reads, and 415 Gb Hi-C data (combined from a leaf and a bud sample). DNA was extracted from a single individual for DNA sequencing except for Hi-C experiments (see below). (**2**) For assembling, we started by selecting 200 Gb, the longest PacBio reads. This subset of reads was corrected using MECAT2, and further trimmed and assembled using CANU version 2.0. The draft assembly was then polished by short reads three rounds using Pilon version 1.23. Finally, chromosome conformation capture (Hi-C) was used to scaffold the contigs using the Juicer, JuiceBox, 3D-DNA pipeline. More details can be found in Methods and Supplementary methods. (**3**) For gene annotation, we collected PacBio Iso-seq sequencing data from 5 tissues, including leaf, bud, pulp, placenta, and root. Each tissue sample was harvested and merged from 5 individual plants. Gene models were predicted using the MAKER pipeline, integrating evidence including full-length transcript isoforms (built by SMRTlink8.0) in 5 tissues obtained from the PacBio Iso-seq method, and gene models from a previous pepper accession, Zunla-1. Transposable elements were predicted by the EDTA pipeline. (4) For the architecture of 3D genome inference, we collected Hi-C data from 4 tissues, including leaf, bud, pulp, and placenta, each with two biology replicates.

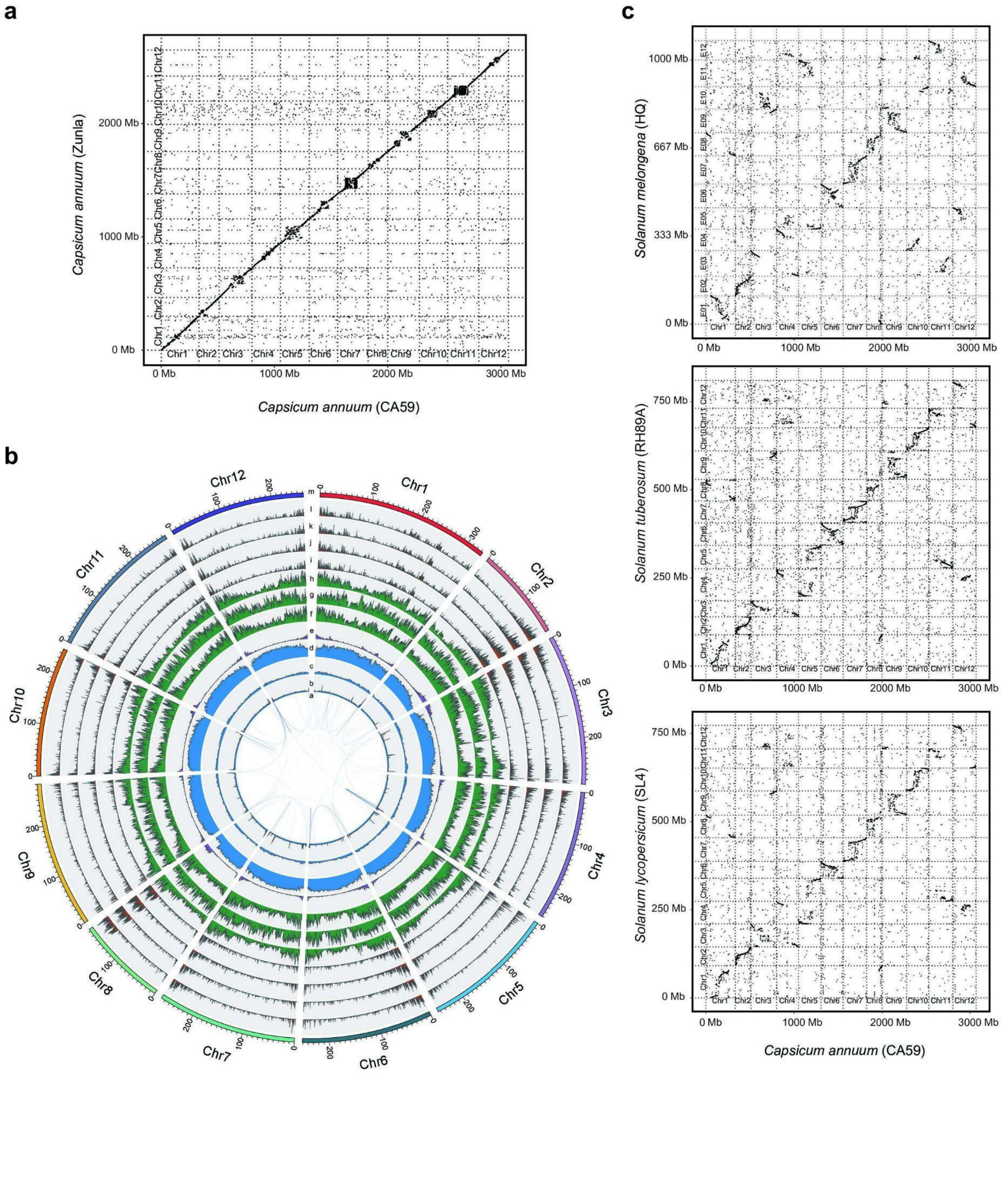

a
b
c
Capsicum annuum (Zunla)
Capsicum annuum (CA59)
0 Mb
1000 Mb
2000 Mb
3000 Mb
Chr1 Chr2 Chr3 Chr4 Chr5 Chr6 Chr7 Chr8 Chr9 Chr10 Chr11 Chr12
Solanum melongena (HQ)
0 Mb
333 Mb
667 Mb
1000 Mb
E01 E02 E03 E04 E05 E06 E07 E08 E09 E10 E11 E12
Solanum tuberosum (RH89A)
0 Mb
250 Mb
500 Mb
750 Mb
Solanum lycopersicum (SL4)
0 Mb
250 Mb
500 Mb
750 Mb
Capsicum annuum (CA59)

Supplementary Fig. 4. Genomic features of the CA59 genome and its synteny with other closely related genomes. **a,** Syntenic dot plot between the *C. annuum* cv. CA59 and cv. Zunla-1 assemblies. **b,** A circos diagram showing the distribution of genomic features. a-e: intra-genome duplications, simple repeats, DNA transposons, LTR retrotransposons, Genes; f-h: SNPs, InDels, SVs (>50bp) identified from five closely related genomes (see Fig. 6a); i-l: transcription profiles in leaf, bud, pulp, and placenta. **c,** Syntenic dot plot between the *C. annuum* cv. CA59 assembly and genomes of three more distantly related Solanaceae species, including eggplant (*S. melongena*), potato (*S. tuberosum*), and tomato (*S. lycopersicum*).

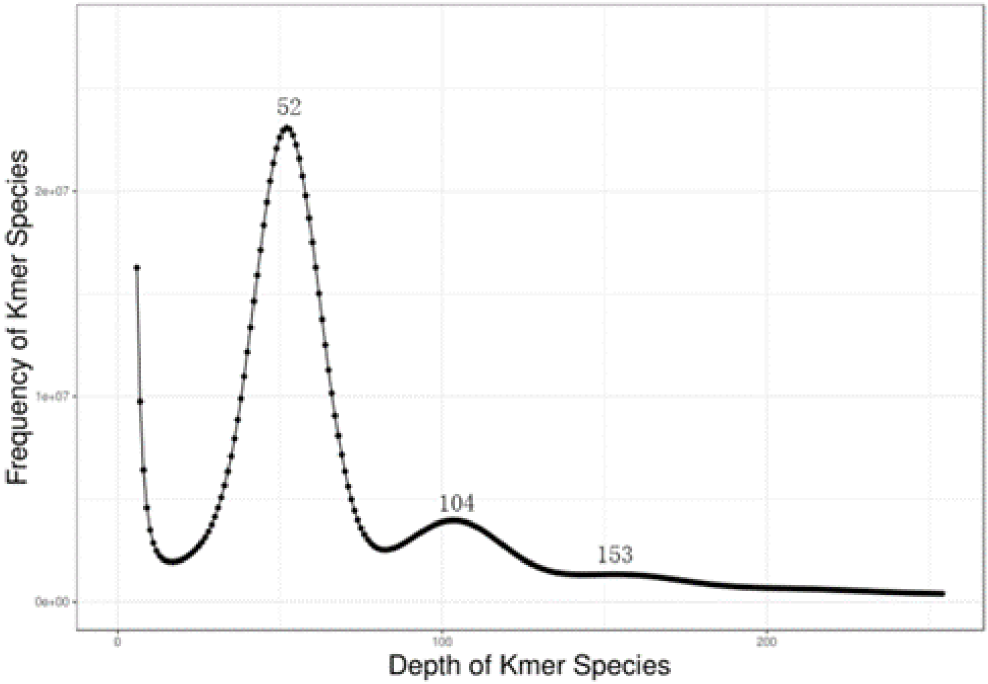

Supplementary Fig. 5. 17-Kmer depth and distribution of the CA59 Short genomic reads. Genome size of CA59 was estimated based on the formula: Total number of Kmer number/Kmer Depth=157,595,048,136/52=3,030,674,003 bp.

a

Intact LTR elements identified per haploid genome in some Solanaceae plants and maize

Species	Genome size (Mb)	Num. of intact elements	Genome occupancy
Pepper	3,077.7	7,074	1.95% (59.89 / 3077.7 Mb)
Tomato	782.5	3,413	3.31% (25.9 / 782.5 Mb)
Eggplant	1,073.1	4,208	3.40% (36.5 / 1,073.1 Mb)
Potato	810.1	3,357	3.28% (26.6 / 810.1 Mb)
Maize	2,131.8	51,980	23.92% (509.9 / 2,131.8 Mb)

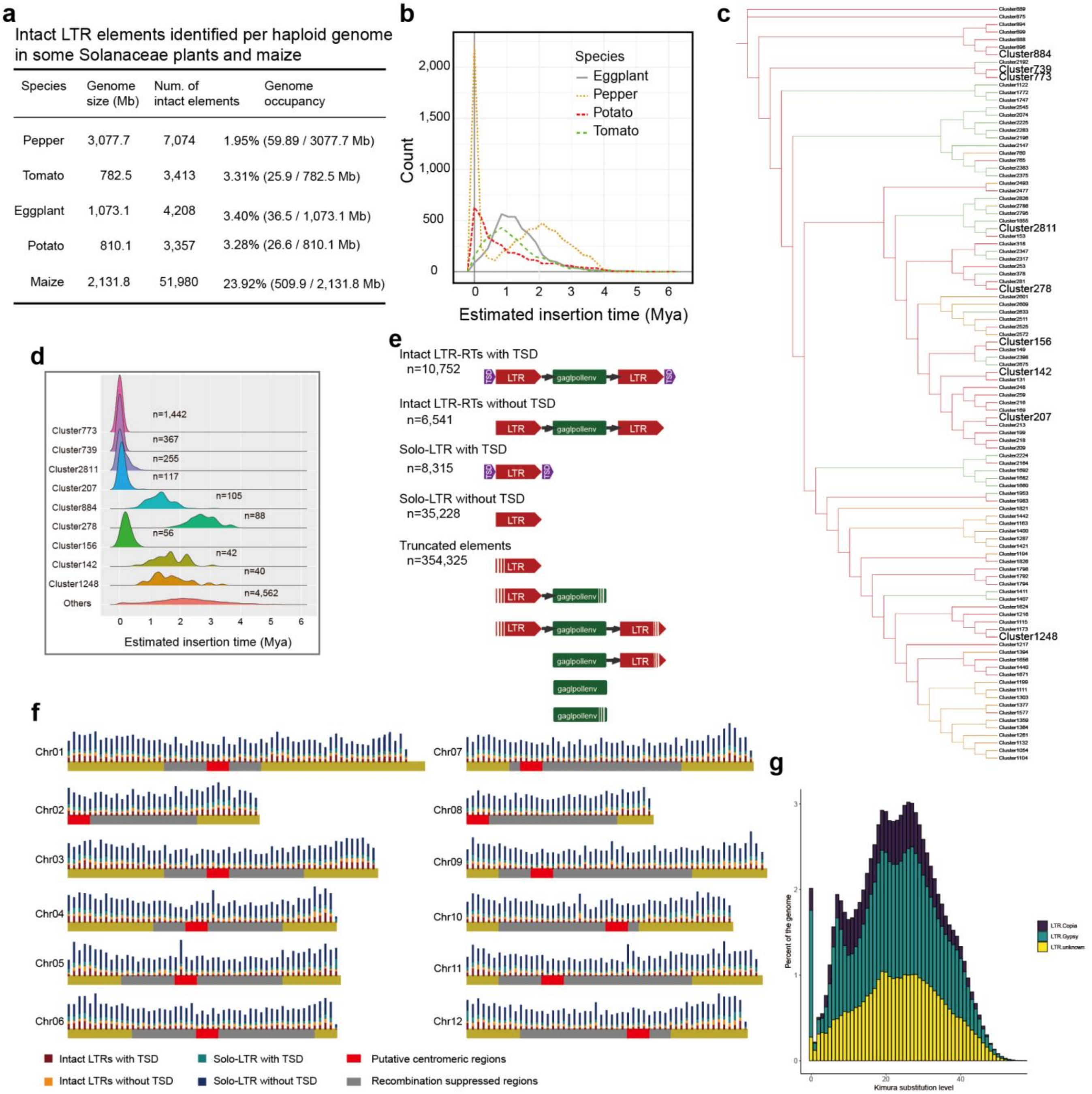

Supplementary Fig. 6. Analysis of LTR-RTs in the CA59 genome assembly. **a,** Sequence occupancy of the intact LTR retrotransposons (LTR-RTs) in the genome of the four Solanaceae species, including pepper (CA59), tomato (SL4), eggplant (HQ), and potato (RH89A), together with maize (B73). **b,** Distribution of the estimated insertion times of intact LTR-RTs in the genome of each Solanaceae species. **c,** Phylogenetic relationship of the top 50 most prominent LTR-RT families in the CA59 genome. Red branches indicate the *gypsy* family, green indicates the *Copia* family, and orange indicates the undetermined family. **d,** Estimated insertion time of the top 9 most prominent LTR-RT families in the CA59 genome. Of them, five families, including clusters 773, 739, 2811, 207, and 156, totaling 2,102 copies, with estimated insertion times almost near zero, indicating they derived from very recent bursts of retroposition. **e,** Schematic representation of the structure of LTR-retrotransposon elements. **f,** Distribution of intact LTR-RTs, solo-LTRs, and fragmented segments along the chromosomes. Categories were summarized for each 5-Mb window. 'TSD' stands for target site duplication. The centromere positions and recombination suppressed regions are taken from a previous work[25]. **g,** The plot of Kimura distance among pairwise alignments between TE sequences identified from RepeatMasker.

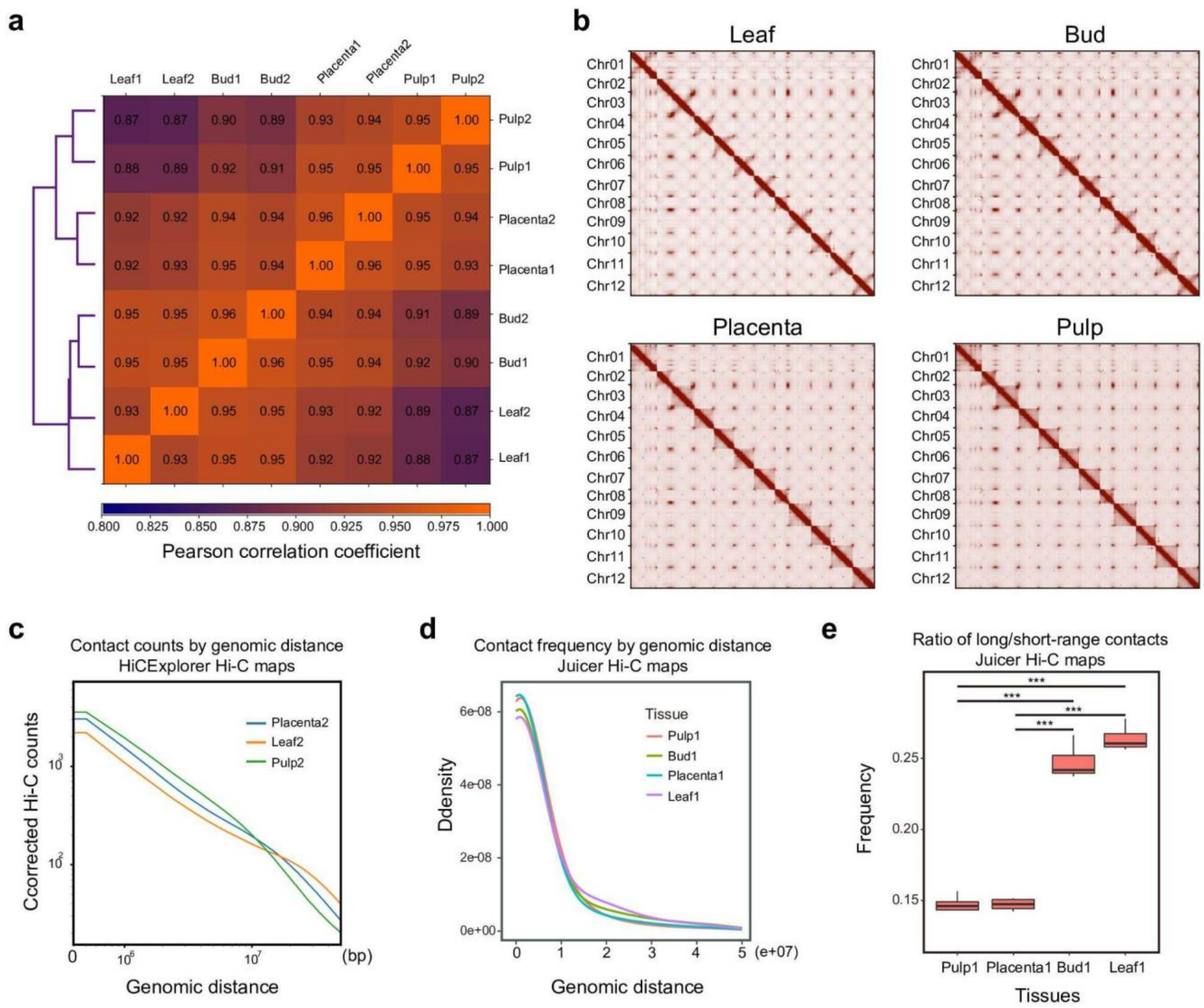
a
Leaf1
Leaf2
Bud1
Bud2
Placenta1
Placenta2
Pulp1
Pulp2
Pearson correlation coefficient
b
Leaf
Bud
Placenta
Pulp
c
Contact counts by genomic distance
HiCExplorer Hi-C maps
Ccorrected Hi-C counts
Genomic distance
(bp)
d
Contact frequency by genomic distance
Juicer Hi-C maps
Ddensity
Tissue
Genomic distance
(e+07)
e
Ratio of long/short-range contacts
Juicer Hi-C maps
Frequency
Tissues

Supplementary Fig. 7. Comparison of Hi-C maps across tissues of pepper. a, Pearson correlation analysis of the corrected Hi-C matrices generated by HiCExplorer at 500 kb resolution across samples. **b**, Genome-wide Hi-C heatmaps generated by juicer at 100-kb resolution across four tissues (supplement to Fig. 1a). **c**, Plot of genomic distance *vs.* contact counts for Hi-C matrices (HiCExplorer) at 500kb resolution. Only samples (leaf, pulp, and placenta) from the second batch were shown here (supplement to Fig. 1c). **d**, Plot of genomic distance *vs.* contact counts for Hi-C matrices at 500kb resolution generated by juicer pipeline. **e**, The ratio of long-range (>20 Mb) versus short-range contacts. Hi-C matrices generated by Juicer were calculated for each chromosome (supplement to Fig. 1d). Box plot shows a median with (the first and third) quartiles. Whiskers extend to 1.5 times IQR. The sample size is n=12 (chromosome number). *** indicates $p < 0.0001$, which was determined by two-side Wilcoxon matched-pairs signed-rank tests. Source Data underlying Supplementary Fig. 7e is provided as a Source Data file.

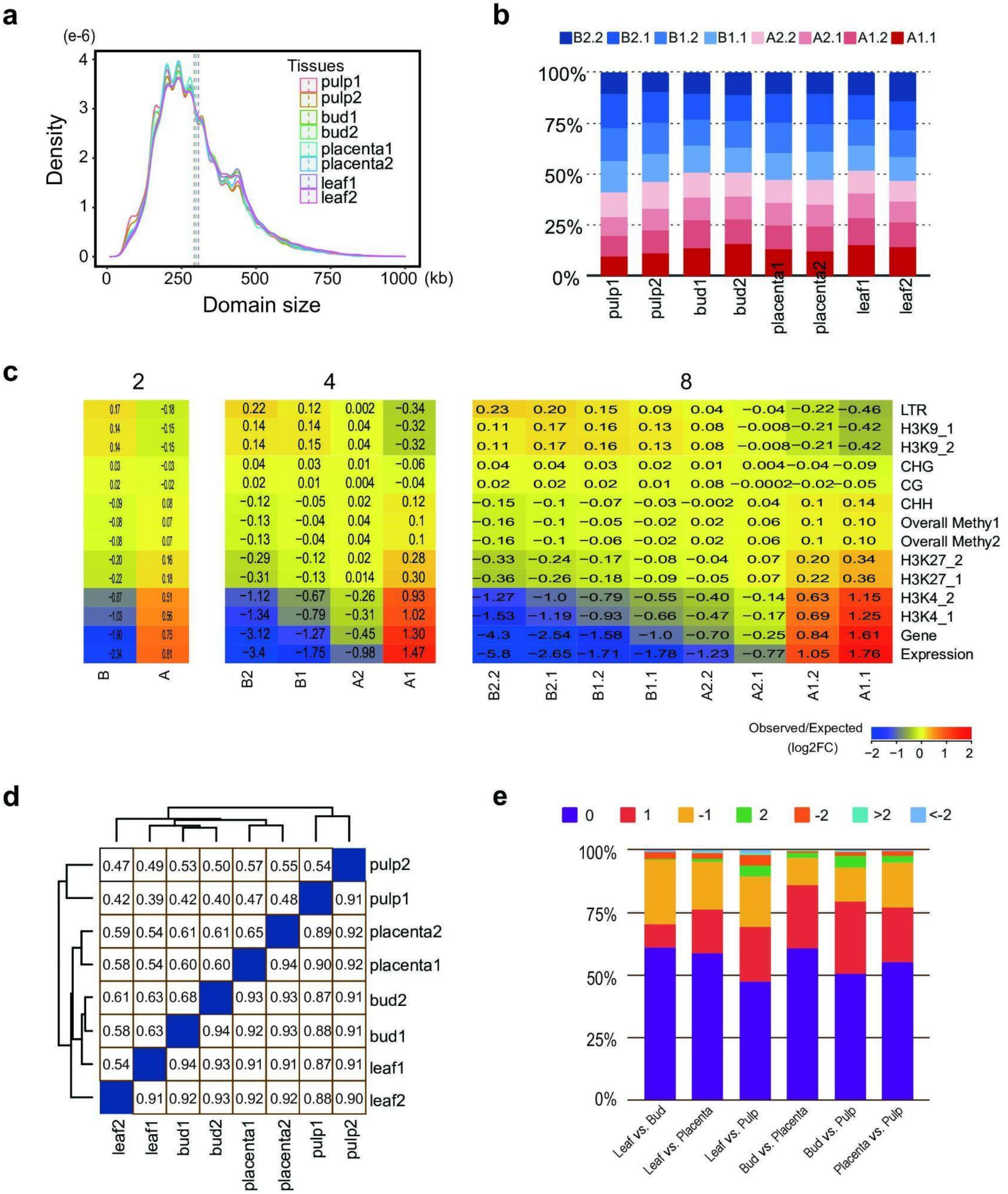
a
(e-6)
Density
Domain size
0 250 500 750 1000 (kb)
0 1 2 3 4
Tissues
pulp1
pulp2
bud1
bud2
placenta1
placenta2
leaf1
leaf2
b
B2.2 B2.1 B1.2 B1.1 A2.2 A2.1 A1.2 A1.1
100% 75% 50% 25% 0%
pulp1 pulp2 bud1 bud2 placenta1 placenta2 leaf1 leaf2
c
2
4
8
0.17 -0.18
0.14 -0.15
0.14 -0.15
0.03 -0.03
0.02 -0.02
-0.09 0.08
-0.08 0.07
-0.08 0.07
-0.20 0.16
-0.22 0.18
-0.87 0.51
-1.03 0.56
-1.90 0.75
-2.34 0.81
B A
0.22 0.12 0.002 -0.34
0.14 0.14 0.04 -0.32
0.14 0.15 0.04 -0.32
0.04 0.03 0.01 -0.06
0.02 0.01 0.004 -0.04
-0.12 -0.05 0.02 0.12
-0.13 -0.04 0.04 0.1
-0.13 -0.04 0.04 0.1
-0.29 -0.12 0.02 0.28
-0.31 -0.13 0.014 0.30
-1.12 -0.67 -0.26 0.93
-1.34 -0.79 -0.31 1.02
-3.12 -1.27 -0.45 1.30
-3.4 -1.75 -0.98 1.47
B2 B1 A2 A1
0.23 0.20 0.15 0.09 0.04 -0.04 -0.22 -0.46 LTR
0.11 0.17 0.16 0.13 0.08 -0.008 -0.21 -0.42 H3K9_1
0.11 0.17 0.16 0.13 0.08 -0.008 -0.21 -0.42 H3K9_2
0.04 0.04 0.03 0.02 0.01 0.004 -0.04 -0.09 CHG
0.02 0.02 0.02 0.01 0.08 -0.0002 -0.02 -0.05 CG
-0.15 -0.1 -0.07 -0.03 -0.002 0.04 0.1 0.14 CHH
-0.16 -0.1 -0.05 -0.02 0.02 0.06 0.1 0.10 Overall Methy1
-0.16 -0.1 -0.06 -0.02 0.02 0.06 0.1 0.10 Overall Methy2
-0.33 -0.24 -0.17 -0.08 -0.04 0.07 0.20 0.34 H3K27_2
-0.36 -0.26 -0.18 -0.09 -0.05 0.07 0.22 0.36 H3K27_1
-1.27 -1.0 -0.79 -0.55 -0.40 -0.14 0.63 1.15 H3K4_2
-1.53 -1.19 -0.93 -0.66 -0.47 -0.17 0.69 1.25 H3K4_1
-4.3 -2.54 -1.58 -1.0 -0.70 -0.25 0.84 1.61 Gene
-5.8 -2.65 -1.71 -1.78 -1.23 -0.77 1.05 1.76 Expression
B2.2 B2.1 B1.2 B1.1 A2.2 A2.1 A1.2 A1.1
Observed/Expected
(log2FC)
-2 -1 0 1 2
d
0.47 0.49 0.53 0.50 0.57 0.55 0.54 pulp2
0.42 0.39 0.42 0.40 0.47 0.48 0.91 pulp1
0.59 0.54 0.61 0.61 0.65 0.89 0.92 placenta2
0.58 0.54 0.60 0.60 0.94 0.90 0.92 placenta1
0.61 0.63 0.68 0.93 0.93 0.87 0.91 bud2
0.58 0.63 0.94 0.92 0.93 0.88 0.91 bud1
0.54 0.94 0.93 0.91 0.91 0.87 0.91 leaf1
0.91 0.92 0.93 0.92 0.92 0.88 0.90 leaf2
leaf2 leaf1 bud1 bud2 placenta1 placenta2 pulp1 pulp2
e
0 1 -1 2 -2 >2 <-2
100% 75% 50% 25% 0%
Leaf vs. Bud
Leaf vs. Placenta
Leaf vs. Pulp
Bud vs. Placenta
Bud vs. Pulp
Placenta vs. Pulp

Supplementary Fig. 8. Characterization of subcompartments (inferred from Hi-C maps at 40-kb resolution) in the pepper genome. a, The size distribution of the *Calder*-inferred subcompartments across tissues. All samples display a constant size distribution with a mean value of ~300kb. **b**, The percentage of each Calder-inferred subcompartment (e.g. A1.1, A1.2, A2.1, A2.2, B1.1, B1.2, B2.1, and B2.2) across tissues. A and B compartments each occupy roughly half of the genome. **c**, Subcompartments are correlated with a number of genomic and epigenomic features. **d**, Similarity of the A/B compartments and subcompartments between tissues. The upper part of the matrix is shown for subcompartments, and the lower part of the matrix is shown for A/B compartments. **e**, Subcompartment switching across four tissues. Pairwise comparisons across four tissues were shown. Numbers above where '0' indicates unchanged subcompartment, '1', '2', and '>2' indicate subcompartment shift spanning 1, 2 or more than 2 subcompartments for lower ranks to higher ranks, and '-1', '-2', and '<-2' indicate subcompartment shift spanning 1, 2 or more than 2 subcompartments for higher ranks to lower ranks. Source Data underlying Supplementary Fig. 8a,b is provided as a Source Data file.

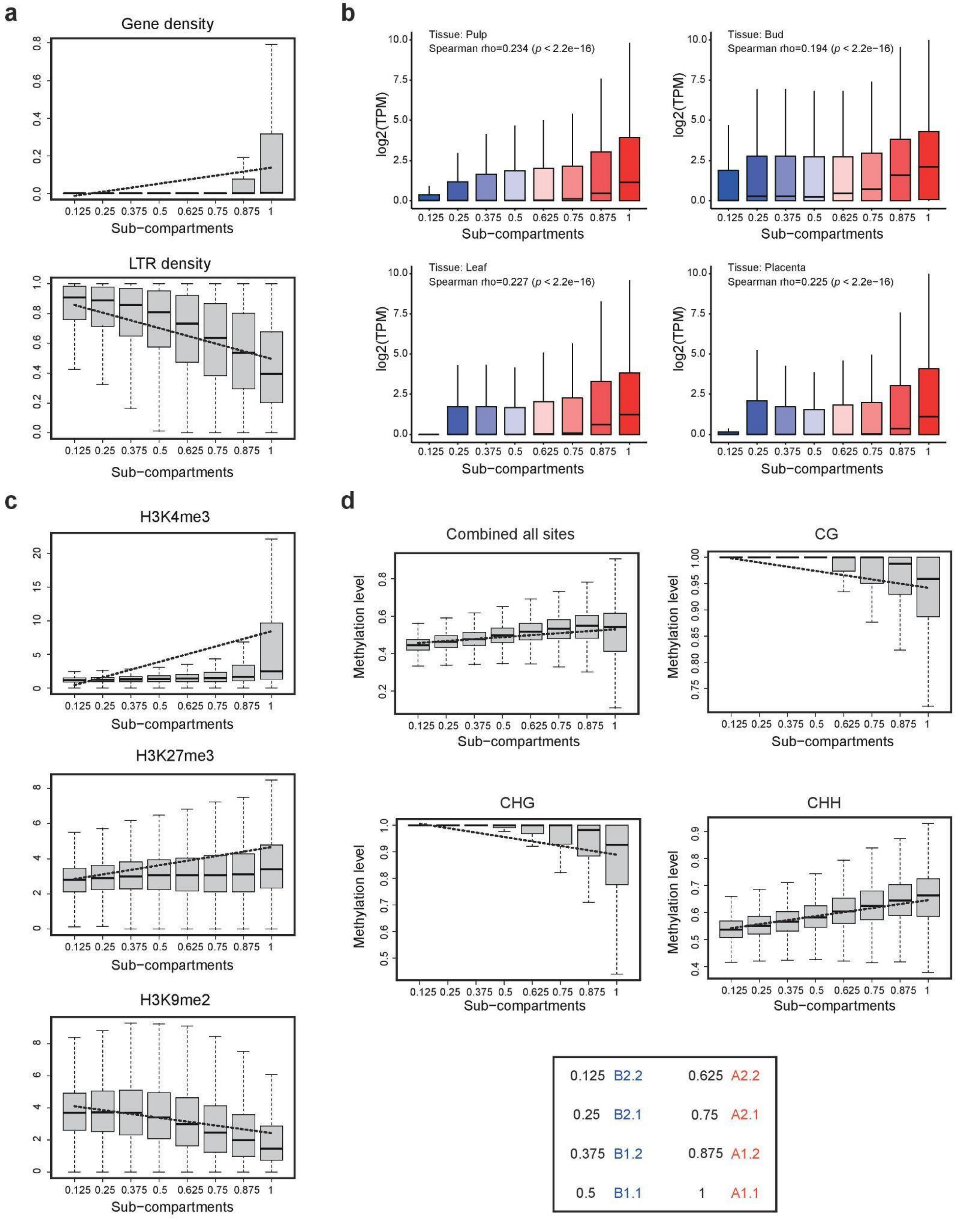
a
Gene density
LTR density
Sub−compartments
b
Tissue: Pulp
Spearman rho=0.234 (p < 2.2e−16)
Tissue: Bud
Spearman rho=0.194 (p < 2.2e−16)
Tissue: Leaf
Spearman rho=0.227 (p < 2.2e−16)
Tissue: Placenta
Spearman rho=0.225 (p < 2.2e−16)
log2(TPM)
Sub−compartments
c
H3K4me3
H3K27me3
H3K9me2
Sub−compartments
d
Combined all sites
CG
CHG
CHH
Methylation level
Sub−compartments
0.125 0.25 0.375 0.5 0.625 0.75 0.875 1
0.125 B2.2
0.625 A2.2
0.25 B2.1
0.75 A2.1
0.375 B1.2
0.875 A1.2
0.5 B1.1
1 A1.1

Supplementary Fig. 9. Subcompartments are correlated with a number of genomic and epigenomic features in the pepper genome. **a**, Calder-inferred subcompartment ranks are positively correlated with gene density (up) but negatively correlated with LTR-RTs density (low). **b**, Subcompartment ranks are positively correlated with transcription levels. The box plot includes a median with (the first and third) quartiles and whiskers. *P*-values were calculated for Spearman's rank correlation. **c**, Subcompartments are correlated with histone modifications. **d**, Correlations between subcompartment ranks and DNA methylation level. "Combined all sites" indicates all CG and non-CG (i.e. CHG and CHH) sites. The box plots in (**a**), (**c**), and (**d**) span from the 25th to 75th percentile, the center lines show the median, and whiskers show maximum and minimum values. The number of 10-kb bins from subcompartment 0.125 to 1 are 77505, 53291, 36007, 30038, 26587, 25986, 24789, and 29311. Dashed lines represent the fitted linear regression curves.

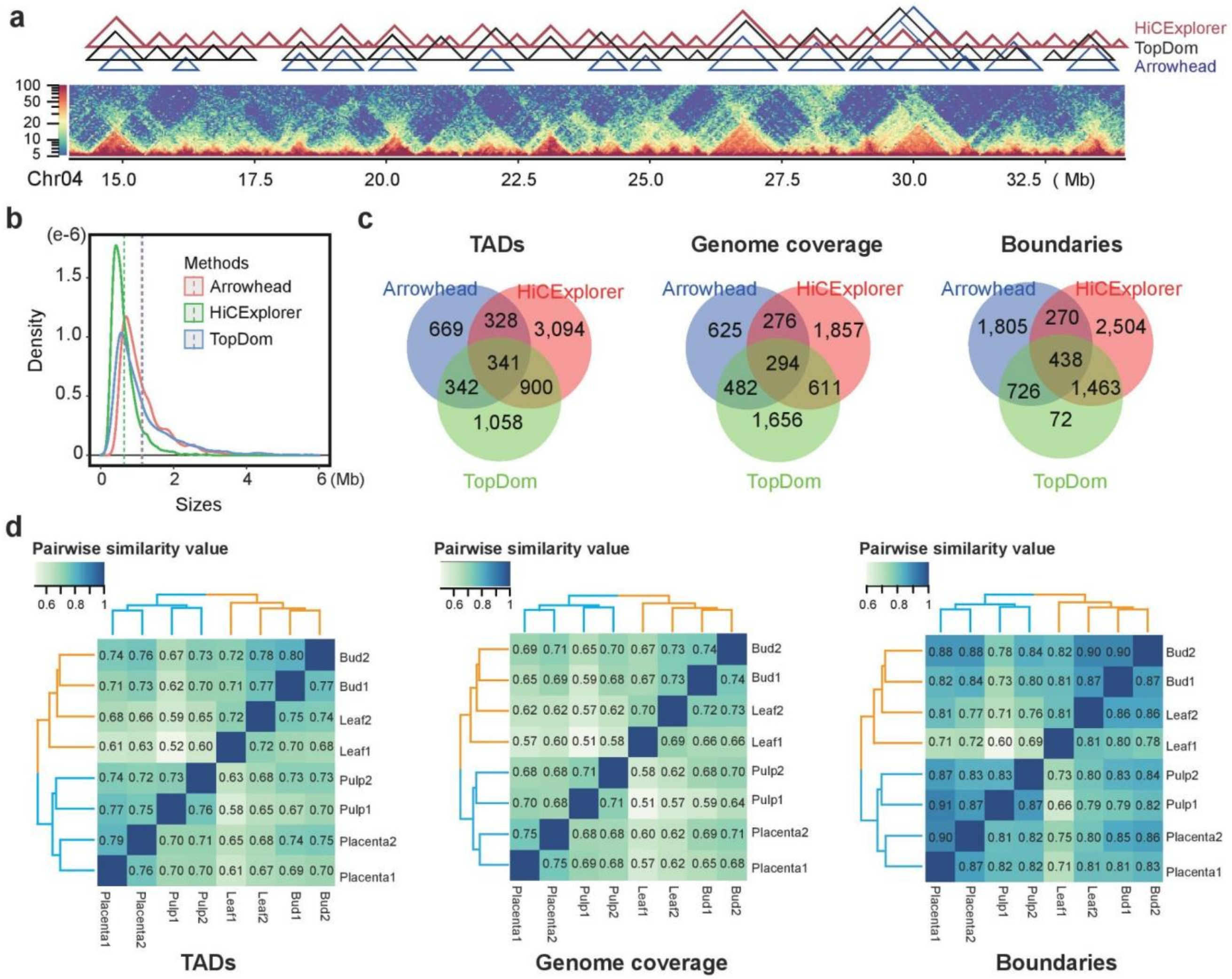

Supplementary Fig. 10. Topologically associating domains annotated using different programs, and similarity of TAD structures across tissues. **a,** Example of TAD annotation for a 20-Mb region on chromosome 4. TADs were annotated by HiCExplorer, TopDom, and Arrowhead using a leaf Hi-C map at 40-kb resolution. **b,** The size distribution of TADs identified by different programs. The mean values were indicated by dashed lines. **c,** Overlap of TADs annotated by different programs, measured in TADs (number), genome coverage, and TAD boundaries. **d,** Hierarchical clustering of samples based on their similarity of TADs, genome coverage, and TAD boundaries. For analyses in **a,b,c**, TAD were annotated from the leaf Hi-C map at 40-kb resolution. For the analysis in **d**, we took TADs from TopDom based on *BNBC* corrected Hi-C maps. Source Data underlying Supplementary Fig. 10b,c is provided as a Source Data file.

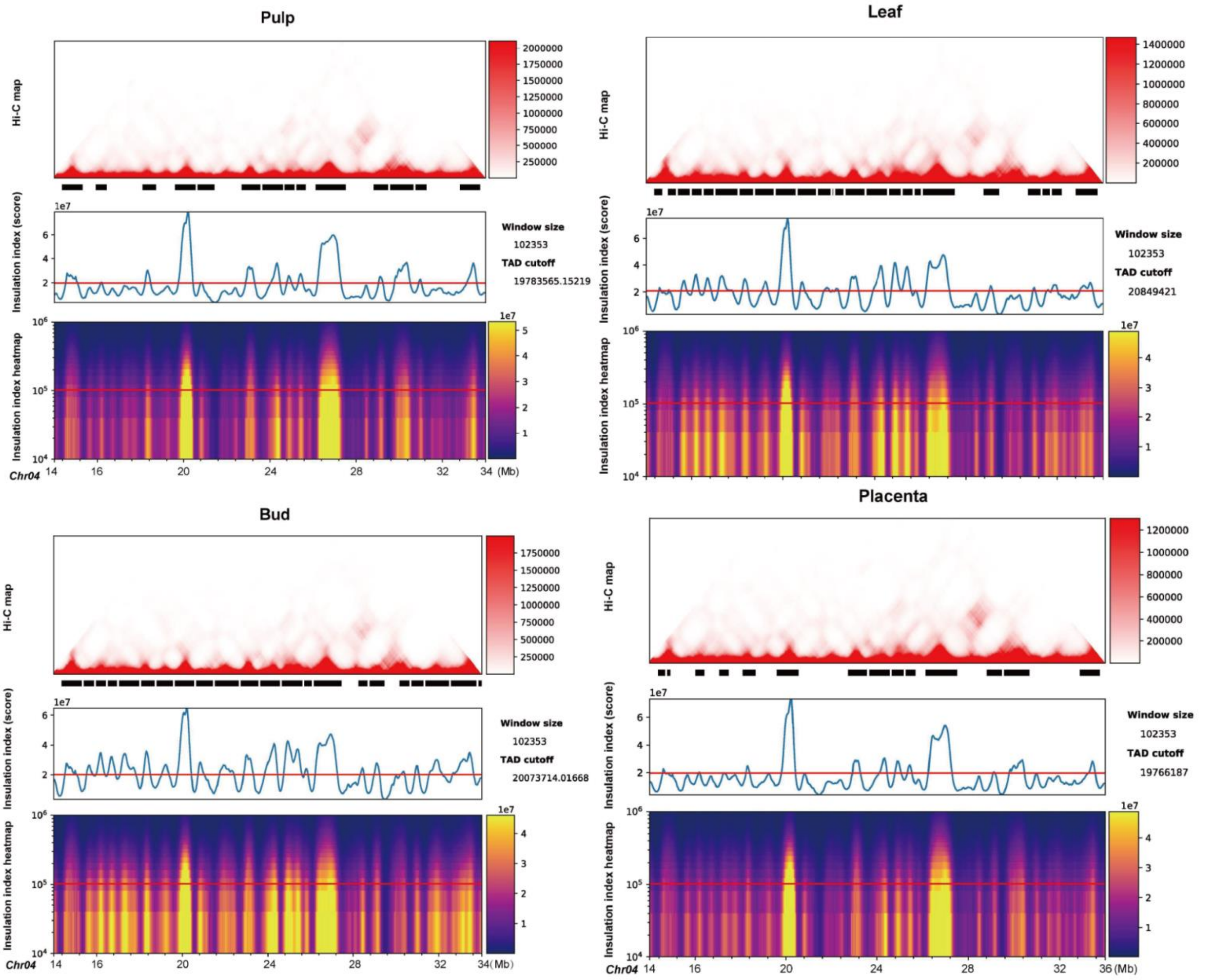

Supplementary Fig. 11. **TAD-like domains inferred by TADtool**. An example region (Chr04: 14,000,000-34,000,000) was shown for four studied tissues. For each tissue, a Hi-C plot, the inferred TADs (black bars), insulation index plot for current window size, and heatmap of insulation index for all window sizes of this region were presented.

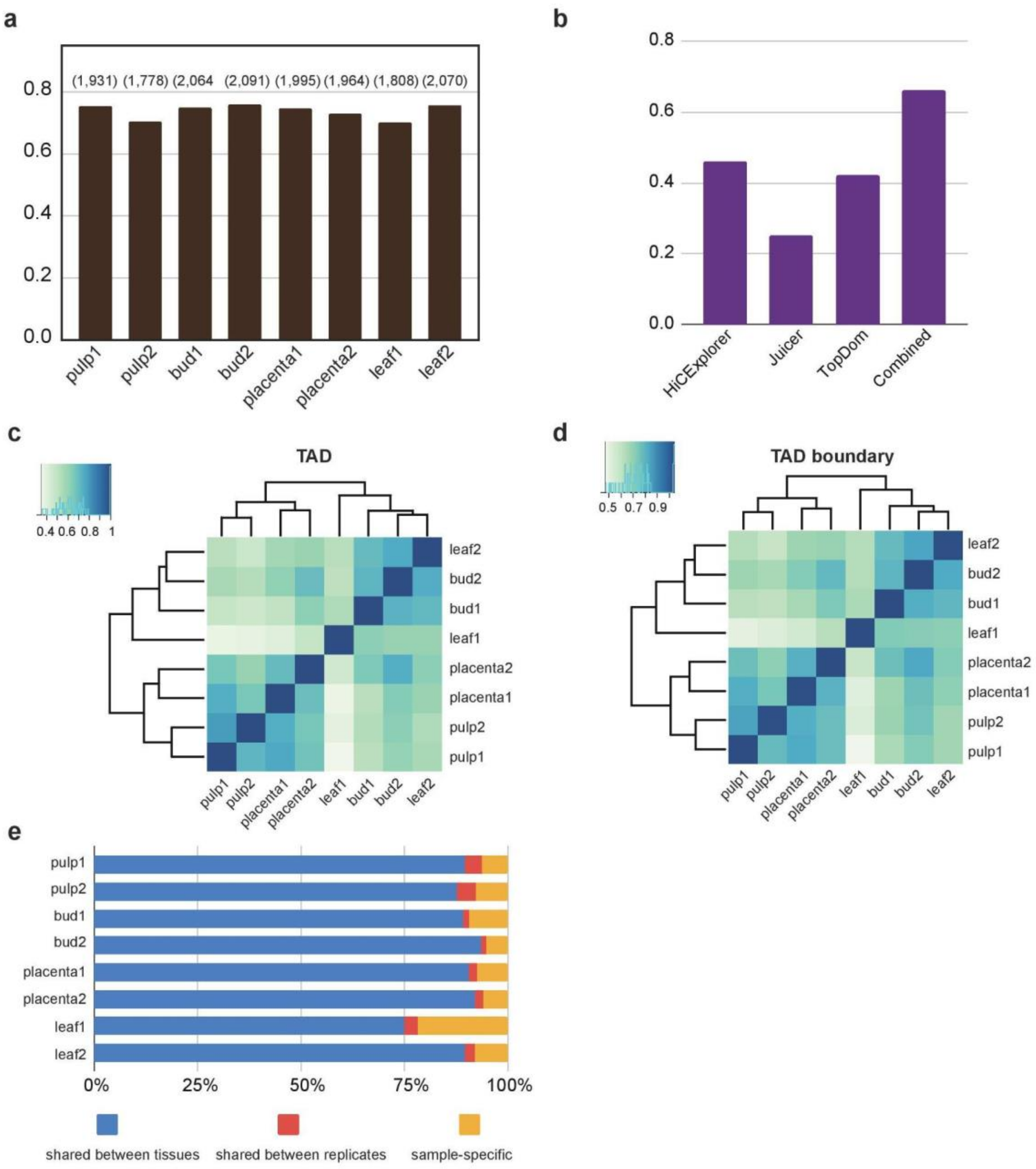

Supplementary Fig. 12. Analysis of TAD-like domains inferred by TADtool. a, Number and genome coverage of domains for all samples. **b,** Overlap of TAD-like domains inferred by TADtool between the other three methods. **c,d,** Hierarchical clustering analysis of the called domains based on the conservation of TADs and boundaries across tissues and biological replicates. As expected, tissues are generally clustered together. **e**, Conservation of domains across tissues. Domains annotated by TADtool at 40 kb resolution were used for this analysis. Source Data underlying Supplementary Fig. 12a is provided as a Source Data file.

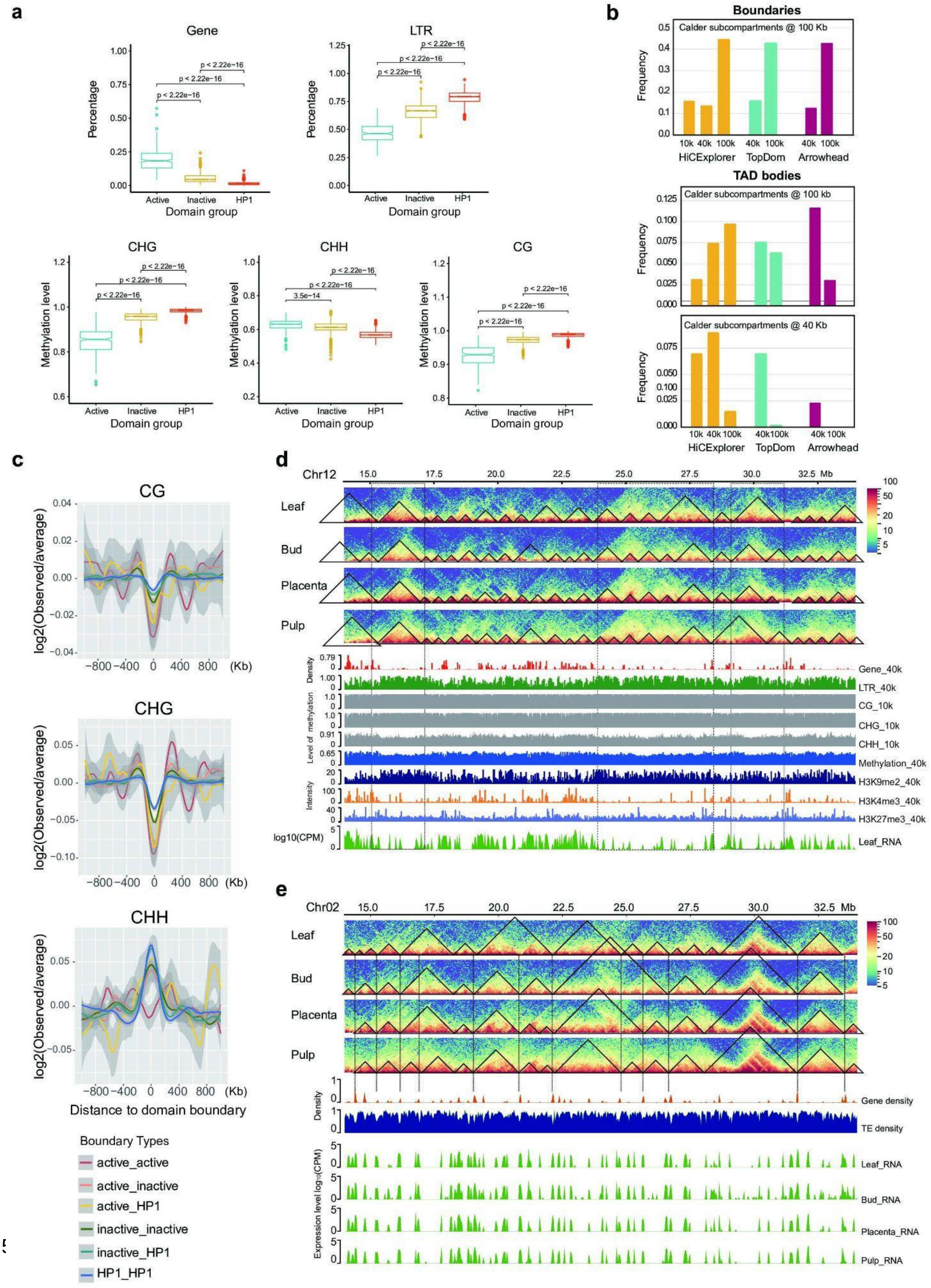

a
Gene
LTR
Percentage
Domain group
Active
Inactive
HP1
p < 2.22e-16
CHG
CHH
CG
Methylation level
3.5e-14
b
Boundaries
Calder subcompartments @ 100 Kb
Frequency
10k 40k 100k
HiCExplorer
TopDom
Arrowhead
TAD bodies
Calder subcompartments @ 100 kb
Calder subcompartments @ 40 Kb
c
log2(Observed/average)
(Kb)
Distance to domain boundary
Boundary Types
active_active
active_inactive
active_HP1
inactive_inactive
inactive_HP1
HP1_HP1
d
Chr12
Mb
Leaf
Bud
Placenta
Pulp
Density
Level of methylation
Intensity
log10(CPM)
Gene_40k
LTR_40k
CG_10k
CHG_10k
CHH_10k
Methylation_40k
H3K9me2_40k
H3K4me3_40k
H3K27me3_40k
Leaf_RNA
e
Chr02
Gene density
TE density
Expression level log10(CPM)
Leaf_RNA
Bud_RNA
Placenta_RNA
Pulp_RNA

Supplementary Fig. 13. Characterization and classification of TAD-like domains in the pepper genome. a, Gene and LTR retrotransposons density for domains of active, inactive, and HFD groups. DNA methylation levels at different contexts (i.e. CHG, CHH, and CpG) for domains of active (n=315), inactive(n=1,011), and HFD groups (n=1,315). The box plot shows a median value with quartiles (25th and 75th) and outliers above or below the top or bottom whiskers. *P* values reported were determined by two-sided Wilcoxon rank-sum tests. **b,** Fraction of the annotated TAD-like domains coincide with compartment/subcompartment domains, so as for boundary. For domain body, subcompartment domains were inferred using *Calder* on HiC maps of 40-kb and 100-kb resolutions. TADs were annotated using HiCExplorer based on Hi-C maps of 10-kb, 40-kb, and 100-kb resolutions, as well as using TopDom and Arrowhead on Hi-C maps of 40-kb and 100-kb resolutions. For boundaries, we compared *Calder*-inferred subcompartments on Hi-C maps of 100-kb resolution to TADs annotated through HiCExplorer on Hi-C maps of 10-kb, 40-kb, and 100-kb resolutions, as well as that annotated through both TopDom and Arrowhead on Hi-C maps of 40-kb and 100-kb resolutions. **c,** DNA methylation level in CpG, CHG, and CHH contexts centered at boundaries of different types. The standard error bounds were computed using the loess method based on a t-based approximation executed in ggplot's smooth geometry in R. **d,** An example of TAD bodies repleting with retrotransposons (above) for a genomic region on chromosome 12, and **e**, an example of TAD boundaries enriched for genes (below) for a genomic region on chromosome 2. Chromatin domains called through HiCExplorer with Hi-C maps at 40-kb resolution were used for analyses in **d-e.**

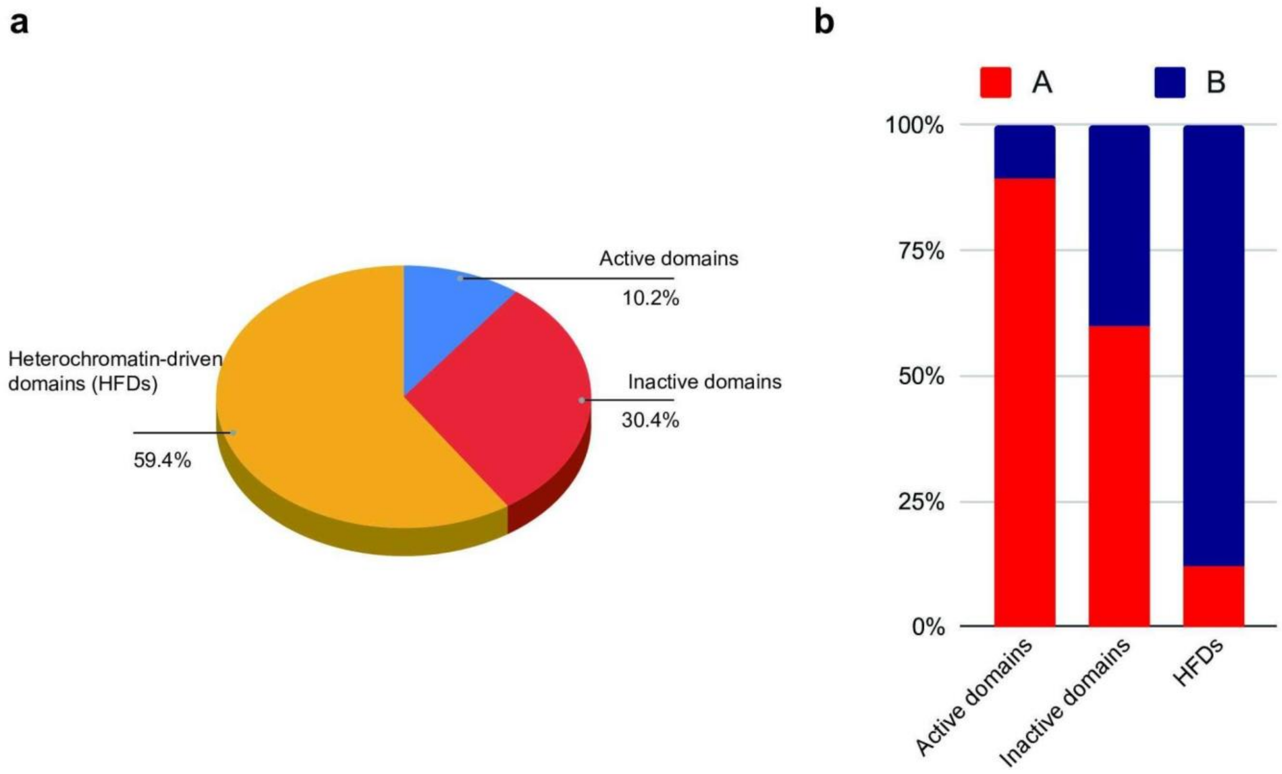

Supplementary Fig. 14. TAD-like domains and their overlapping with ‘AB’ compartments. a, Genome coverage for groups of TAD-like domains. **b,** Overlapping between TAD-like domains and ‘AB’ compartments by groups.

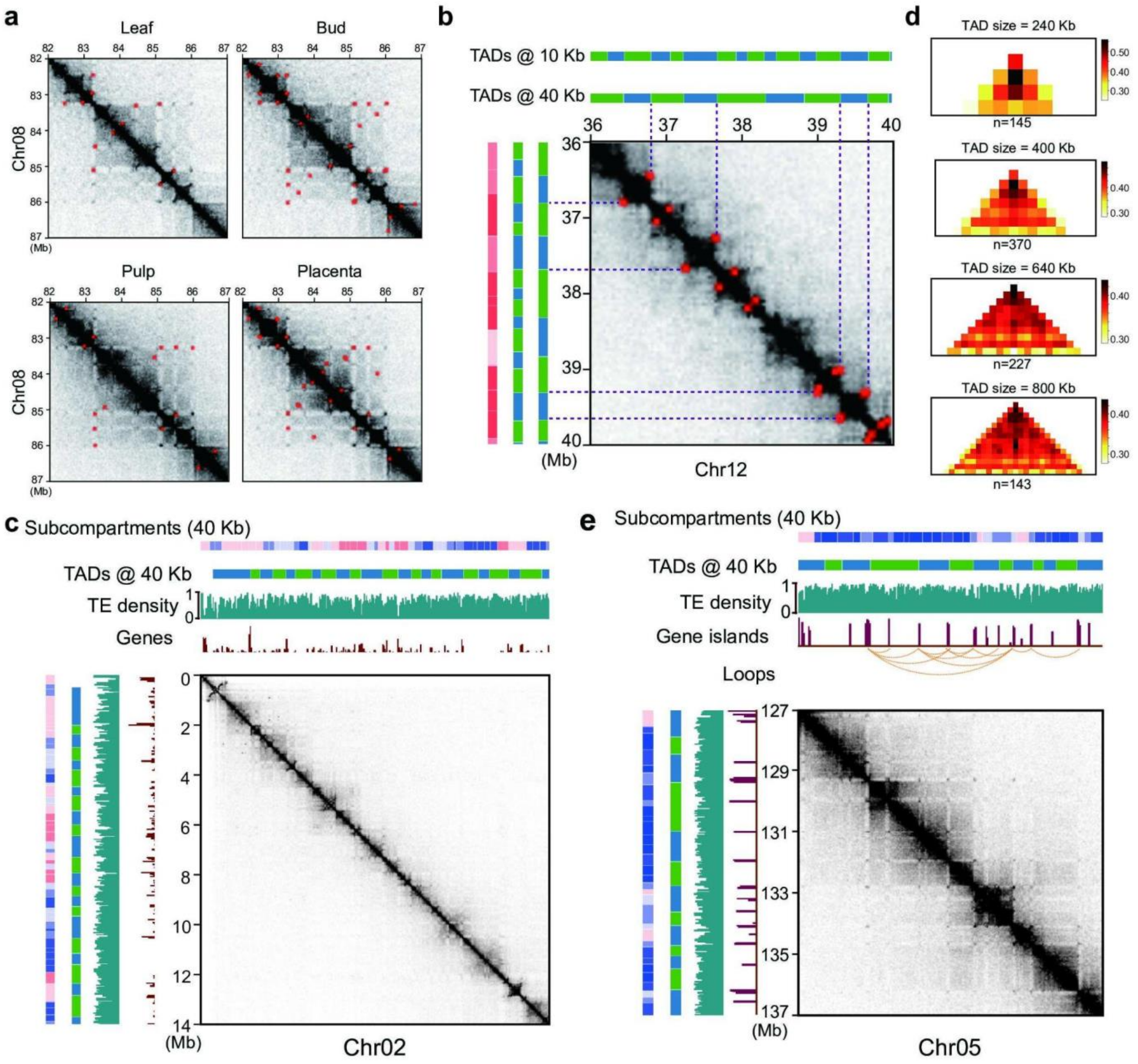
a
Leaf
Bud
Pulp
Placenta
Chr08
(Mb)
b
TADs @ 10 Kb
TADs @ 40 Kb
Chr12
(Mb)
d
TAD size = 240 Kb
n=145
TAD size = 400 Kb
n=370
TAD size = 640 Kb
n=227
TAD size = 800 Kb
n=143
c
Subcompartments (40 Kb)
TADs @ 40 Kb
TE density
Genes
Chr02
e
Subcompartments (40 Kb)
TADs @ 40 Kb
TE density
Gene islands
Loops
Chr05

Supplementary Fig. 15. Chromatin loops in the pepper genome. a, Example of loop annotation for a 5-Mb region on chromosome 8 across four tissues. Loops (indicated in red dots) detected in one tissue were missing in another, likely because they were present but below the threshold of detection. Loops were identified by hicDetectLoops. **b,** Example showing a genomic region (Chr12: 36,000,000 - 40,000,000) where chromatin loops demarcate TADs. Subcompartments and TADs identified at both 10-kb and 40-kb resolution for this region were shown above and right (supplement to Fig. 5c). Loops were shown as red dots in the Hi-C contact maps (leaf 40 kb resolution). Dashed purple lines indicate the coincidence of TAD boundaries and loop anchors. **c,** Enhanced contact frequency between the two corners of TADs. TAD sizes are shown on top. The number of TADs for each size is shown below. TADs are identified in the leaf Hi-C map at 40-kb resolution (supplement to Fig. 5d). **d,** Example of Hi-C map showing TADs are demarcated by loops in a gene-rich region on chromosome 2. **e,** Example of gene-to-gene loops for a 10-kb genomic region on chromosome 5 (supplement to Fig. 5e).

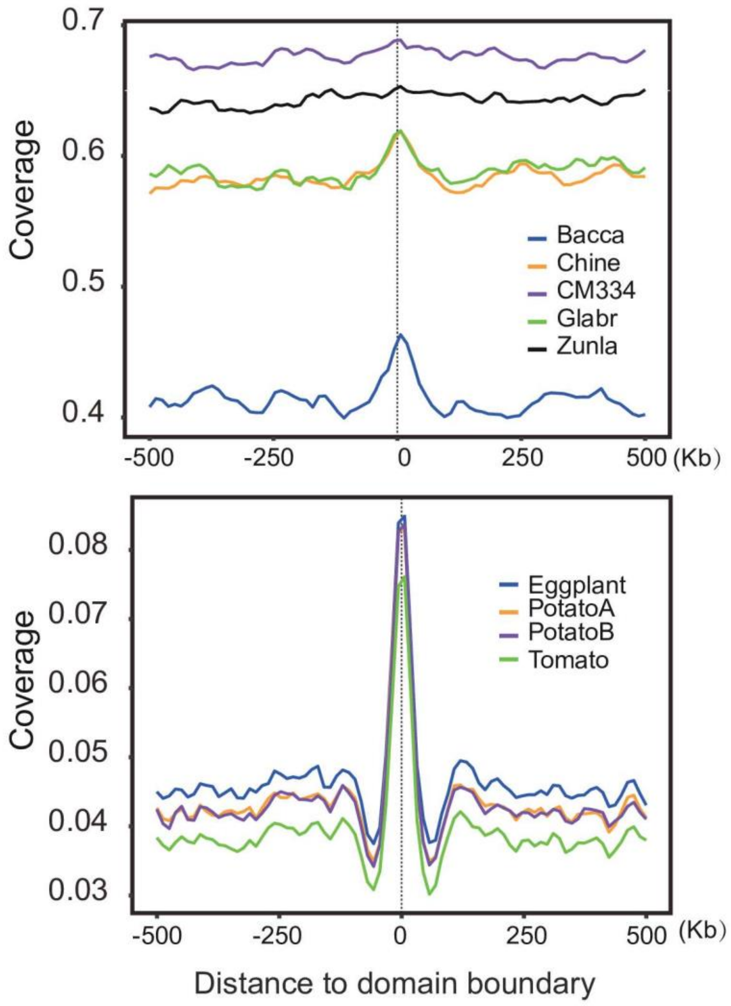

Supplementary Fig. 16. TAD boundaries are enriched for evolutionary sequence conservation (supplement to Fig. 6b). Chromatin domains are called through HiCExplorer with Hi-C maps at 40-kb resolution.

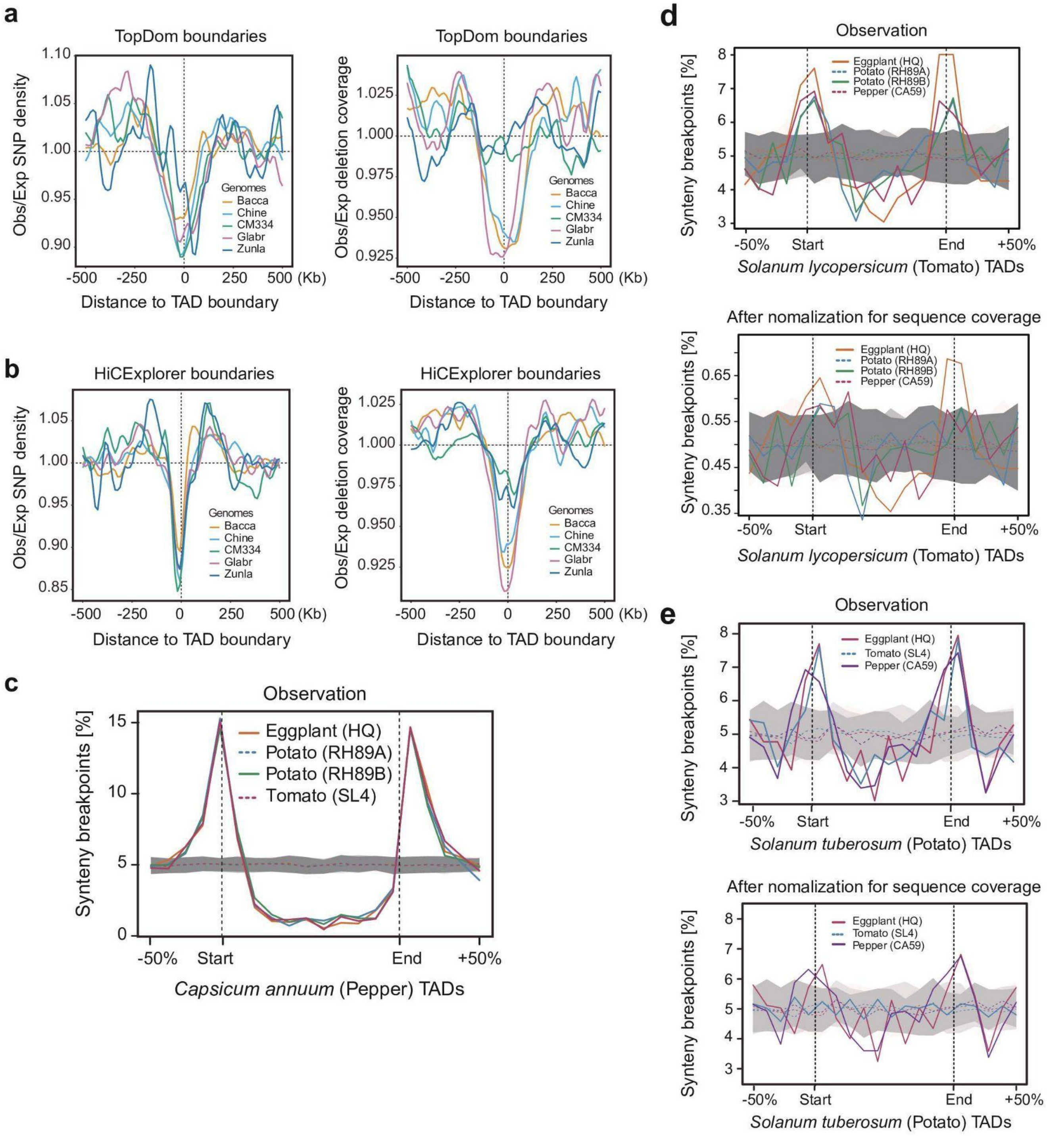

a
TopDom boundaries
Obs/Exp SNP density
Obs/Exp deletion coverage
Distance to TAD boundary
(Kb)
Genomes
Bacca
Chine
CM334
Glabr
Zunla
b
HiCExplorer boundaries
c
Observation
Synteny breakpoints [%]
Eggplant (HQ)
Potato (RH89A)
Potato (RH89B)
Tomato (SL4)
-50%
Start
End
+50%
Capsicum annuum (Pepper) TADs
d
Pepper (CA59)
Solanum lycopersicum (Tomato) TADs
After nomalization for sequence coverage
e
Solanum tuberosum (Potato) TADs

Supplementary Fig. 17. Synteny breaks among genomes of solanaceous species are enriched at TAD boundaries, despite evolutionary conservation. **a,b,** The observed (Obs) distribution of SNPs and deletions (coverage) near TAD boundaries relative to the expectation (Exp), based on the genomic background. SNPs and deletions were identified in five closely related genomes (see Fig. 6a) relative to CA59. TADs were annotated by TopDom (**a**) and HiCExplorer (**b**) using leaf Hi-C data at 40-kb resolution (supplement to Fig. 6c). The expected genomic background was calculated as the mean value of all bined windows within 500 kb downstream and upstream of TAD boundaries. **c,** TAD boundaries (observed) of pepper are enriched for evolutionary synteny breaks identified from distantly related solanaceous species (supplement to Fig. 6f). Dotted lines in gray show randomly simulated synteny breaks (n=100). **d,** TAD boundaries of tomato (*Solanum lycopersicum*) are enriched for evolutionary synteny breaks identified from the other three distantly related solanaceous species (eggplant, tomato, and pepper). The top plot shows the observed values, while the bottom shows the normalized values for evolutionary sequence coverage. TADs were annotated by HiCExplorer using Hi-C maps at 40 kb resolution. **e,** Similar analyses as **d** when using potato (*Solanum tuberosum*) as the reference. Simulated synteny breaks data (n=100) in (**c-e**) are presented as mean ± SD.

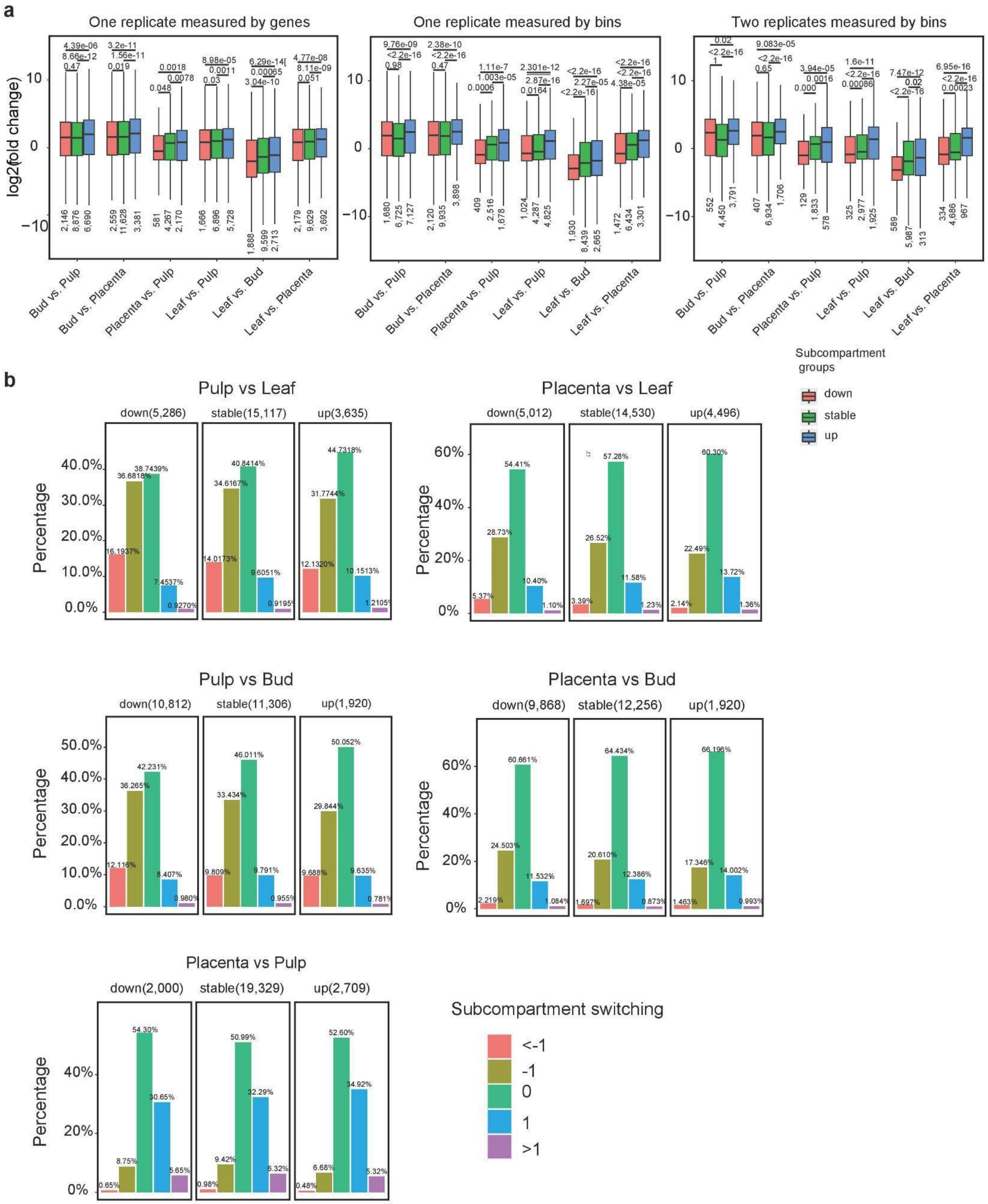
a
One replicate measured by genes
One replicate measured by bins
Two replicates measured by bins
log2(fold change)
10
0
−10
Bud vs. Pulp
Bud vs. Placenta
Placenta vs. Pulp
Leaf vs. Pulp
Leaf vs. Bud
Leaf vs. Placenta
Subcompartment groups
down
stable
up
b
Pulp vs Leaf
down(5,286)
stable(15,117)
up(3,635)
Percentage
Placenta vs Leaf
down(5,012)
stable(14,530)
up(4,496)
Pulp vs Bud
down(10,812)
stable(11,306)
up(1,920)
Placenta vs Bud
down(9,868)
stable(12,256)
up(1,920)
Placenta vs Pulp
down(2,000)
stable(19,329)
up(2,709)
Subcompartment switching
<-1
-1
0
1
>1

Supplementary Fig. 18. The relationship between subcompartment switching and change in gene expression. a, Genomic regions (i.e. 40-kb bins) switching from A to B compartments or from higher subcompartments to lower subcompartments (e.g. from A1.1 to A1.2) show a trend of decreasing expression, and conversely, switching from B to A compartment or from lower subcompartments to higher subcompartments show a trend of increasing expression. Pairwise comparisons of subcompartment shifts for expression profiles across the leaf, bud, pulp, and placenta are shown(supplement to Fig. 7a). Analyses were conducted in two ways: 1) only considered one replicate (i.e. a subcompartment switching event only needed to be supported in the first replicate), and 2) two replicates (i.e. a subcompartment switching event needed to be supported by both replicates). The expression level was measured in genes or 40-kb bins. The box plots span from 25th to 75th percentile, the center lines show the median, and whiskers extend to 1.5 times IQR. The numbers under lower whiskers indicate the sample size used in the analysis. *P* values from one-sided Wilcoxon ranked sum tests. **b,** 40-kb bins with decreased expression were slightly enriched for cases of subcompartment switching from a higher rank to lower ranks, while those with increased expression were slightly enriched for cases of subcompartment switching from lower ranks to higher ranks (supplement to Fig. 7b).

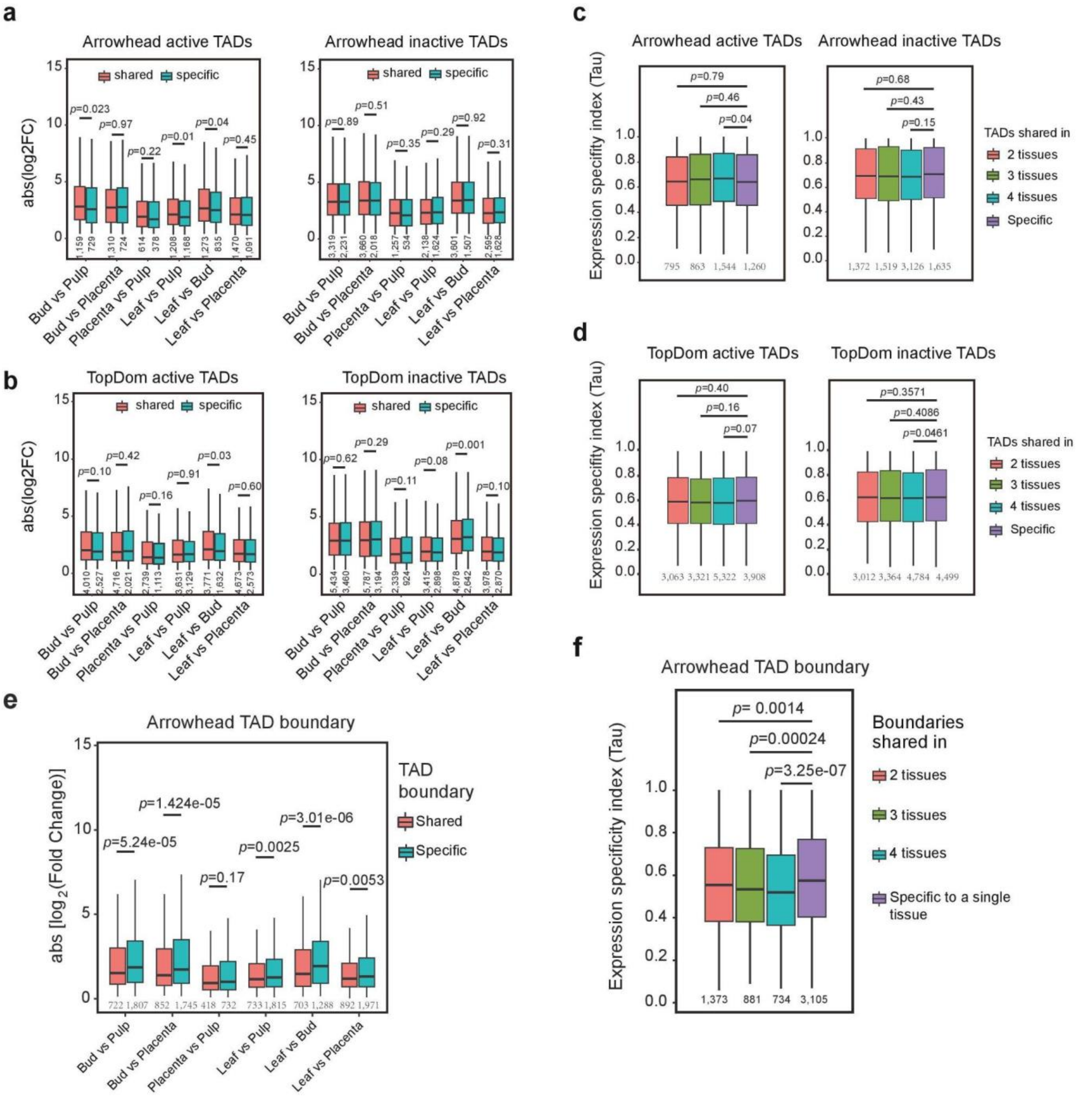
a
Arrowhead active TADs
Arrowhead inactive TADs
shared
specific
abs(log2FC)
p=0.023
p=0.97
p=0.22
p=0.01
p=0.04
p=0.45
p=0.89
p=0.51
p=0.35
p=0.29
p=0.92
p=0.31
Bud vs Pulp
Bud vs Placenta
Placenta vs Pulp
Leaf vs Pulp
Leaf vs Bud
Leaf vs Placenta
b
TopDom active TADs
TopDom inactive TADs
p=0.10
p=0.42
p=0.16
p=0.91
p=0.03
p=0.60
p=0.62
p=0.29
p=0.11
p=0.08
p=0.001
p=0.10
c
Arrowhead active TADs
Arrowhead inactive TADs
Expression specifity index (Tau)
p=0.79
p=0.46
p=0.04
p=0.68
p=0.43
p=0.15
TADs shared in
2 tissues
3 tissues
4 tissues
Specific
795 863 1,544 1,260
1,372 1,519 3,126 1,635
d
TopDom active TADs
TopDom inactive TADs
p=0.40
p=0.16
p=0.07
p=0.3571
p=0.4086
p=0.0461
3,063 3,321 5,322 3,908
3,012 3,364 4,784 4,499
e
Arrowhead TAD boundary
abs [log2(Fold Change)]
TAD boundary
Shared
Specific
p=5.24e-05
p=1.424e-05
p=0.17
p=0.0025
p=3.01e-06
p=0.0053
722 1,807 852 1,745 418 732 733 1,815 703 1,288 892 1,971
f
Arrowhead TAD boundary
Expression specificity index (Tau)
p= 0.0014
p=0.00024
p=3.25e-07
Boundaries shared in
2 tissues
3 tissues
4 tissues
Specific to a single tissue
1,373 881 734 3,105

Supplementary Fig. 19. Conservation of TAD boundaries is associated with transcription stability across tissues but not for TAD bodies. **a,b** Box plot of the absolute fold change [abs($\log_2$FC)] of transcription level (measured in 40-kb bin) between tissues. The 40-kb bins were divided into two groups that-are: belonging to shared TADs and tissue-specific TADs. TADs were identified using Arrowhead (**a**) and TopDom (**b**). TADs are further subdivided into active and inactive groups. **c,d** Box plot of the *Tau* value of 40 kb bins. The 40-kb bins were divided into four groups that-are: belonging to TADs that are shared in 2, 3, and 4 tissues, and specific to a single tissue. TADs were identified using Arrowhead (**c**) and TopDom (**d**). **e,** 40-kb bins overlapping with TAD boundaries (Arrowhead) conserved between tissues exhibit a relatively smaller absolute change fold in expression level than those overlapping with tissue-specific TAD boundaries (supplement to Fig. 7c). Pairwise comparisons across four tissues were shown. **f,** 40-kb bins overlapping with shared TAD boundaries (Arrowhead) across tissues exhibit a significantly lower expression specificity index Tau value compared to those overlapped with tissue-specific TAD boundaries (supplement to Fig. 7d). Box plots in (**a-f**) represent the median (band inside the box), first and third quartiles. Whiskers extend to 1.5 times IQR. The numbers under the lower whiskers indicate the sample size used in the analysis. *P* values from one-sided Wilcoxon ranked sum tests.

Supplemental references

1. Liu, B. *et al.* Estimation of genomic characteristics by analyzing k-mer frequency in *de novo* genome projects. arXiv, *https://doi.org/10.48550/arXiv.1308.2012* (2013).
2. Bolger, A. M., Lohse, M. & Usadel, B. Trimmomatic: a flexible trimmer for Illumina sequence data. *Bioinformatics* **30**, 2114–2120 (2014).
3. Porebski, S., Bailey, L. G. & Baum, B. R. Modification of a CTAB DNA extraction protocol for plants containing high polysaccharide and polyphenol components. *Plant Mol. Biol. Rep.* **15**, 8–15 (1997).
4. Xiao, C.-L. *et al.* MECAT: fast mapping, error correction, and de novo assembly for single-molecule sequencing reads. *Nat. Methods* **14**, 1072–1074 (2017).
5. Koren, S. *et al.* Canu: scalable and accurate long-read assembly via adaptive k-mer weighting and repeat separation. *Genome Res*. **27**, 722-736 (2017).
6. Walker, B. J. *et al.* Pilon: an integrated tool for comprehensive microbial variant detection and genome assembly improvement. *PLoS One* **9**, e112963 (2014).
7. Seppey, M., Manni, M. & Zdobnov, E. M. BUSCO: Assessing Genome Assembly and Annotation Completeness. *Methods Mol. Biol.* **1962**, 227–245 (2019).
8. Langmead, B. & Salzberg, S. L. Fast gapped-read alignment with Bowtie 2. *Nat. Methods* **9**, 357–359 (2012).
9. Garrison, E. & Marth, G. Haplotype-based variant detection from short-read sequencing. *arXiv, https://doi.org/10.48550/arXiv.1207.3907* (2012).
10. Dudchenko, O. *et al.* De novo assembly of the genome using Hi-C yields chromosome-length scaffolds. *Science* **356**, 92–95 (2017).
11. Durand, N. C. *et al.* Juicer Provides a One-Click System for Analyzing Loop-Resolution Hi-C Experiments. *Cell Syst* **3**, 95–98 (2016).
12. Kuo, R. I. *et al.* Illuminating the dark side of the human transcriptome with long read transcript sequencing. *BMC Genomics* **21**, 751 (2020).
13. Kent, W. J., Zweig, A. S., Barber, G., Hinrichs, A. S. & Karolchik, D. BigWig and BigBed: enabling browsing of large distributed datasets. *Bioinformatics* **26**, 2204–2207 (2010).
14. Pertea, G. & Pertea, M. GFF Utilities: GffRead and GffCompare. *F1000Res.* **9**, (2020).
15. Kim, D., Paggi, J. M., Park, C., Bennett, C. & Salzberg, S. L. Graph-based genome alignment and genotyping

with HISAT2 and HISAT-genotype. *Nat. Biotechnol.* **37**, 907–915 (2019).

16. Pertea, M. *et al.* StringTie enables improved reconstruction of a transcriptome from RNA-seq reads. *Nature Biotechnology* **33**, 290–295 (2015).
17. Liao, Y., Smyth, G. K. & Shi, W. featureCounts: an efficient general purpose program for assigning sequence reads to genomic features. *Bioinformatics* **30**, 923–930 (2014).
18. Ou, S. *et al.* Benchmarking transposable element annotation methods for creation of a streamlined, comprehensive pipeline. *Genome Biol.* **20**, 275 (2019).
19. Campbell, M. S., Holt, C., Moore, B. & Yandell, M. Genome Annotation and Curation Using MAKER and MAKER- P. *Curr. Protoc. Bioinformatics* **48**, 188 (2014).
20. Ramírez, F. *et al.* High-resolution TADs reveal DNA sequences underlying genome organization in flies. *Nat. Commun.* **9**, 189 (2018).
21. Devos, K. M., Brown, J. K. M. & Bennetzen, J. L. Genome size reduction through illegitimate recombination counteracts genome expansion in Arabidopsis. *Genome Res.* **12**, 1075–1079 (2002).
22. Tian, Z. *et al.* Do genetic recombination and gene density shape the pattern of DNA elimination in rice long terminal repeat retrotransposons? *Genome Research* **19**, 2221–2230 (2009).
23. Du, J. *et al.* Evolutionary conservation, diversity and specificity of LTR-retrotransposons in flowering plants: insights from genome-wide analysis and multi-specific comparison. *Plant J.* **63**, 584–598 (2010).
24. Ritchie, M. E. *et al.* limma powers differential expression analyses for RNA-sequencing and microarray studies. *Nucleic Acids Res.* **43**, e47 (2015).
25. Qin, C. *et al.* Whole-genome sequencing of cultivated and wild peppers provides insights into Capsicum domestication and specialization. *Proc. Natl. Acad. Sci. U. S. A.* **111**, 5135–5140 (2014).
26. Kim, S. *et al.* Genome sequence of the hot pepper provides insights into the evolution of pungency in Capsicum species. *Nat. Genet.* **46**, 270–278 (2014).
27. Kim, S. *et al.* New reference genome sequences of hot pepper reveal the massive evolution of plant disease-resistance genes by retroduplication. *Genome Biol.* **18**, 210 (2017).
28. Rao, S. S. P. *et al.* A 3D map of the human genome at kilobase resolution reveals principles of chromatin looping. *Cell* **159**, 1665–1680 (2014).
29. Liu, C., Cheng, Y.-J., Wang, J.-W. & Weigel, D. Prominent topologically associated domains differentiate

global chromatin packing in rice from Arabidopsis. *Nat Plants* **3**, 742–748 (2017).

30. Dong, P. *et al.* 3D Chromatin Architecture of Large Plant Genomes Determined by Local A/B Compartments. *Molecular Plant* **10**, 1497–1509 (2017).
31. Chathoth, K. T. & Zabet, N. R. Chromatin architecture reorganization during neuronal cell differentiation in Drosophila genome. *Genome Res.* **29**, 613–625 (2019).
32. Dixon, J. R. *et al.* Chromatin architecture reorganization during stem cell differentiation. *Nature* **518**, 331–336 (2015).
