# Supplementary material for: The 3D architecture of the pepper genome and its relationship to function and evolution
Source: Nat Commun. 2022 Jun 16;13:3479. doi: 10.1038/s41467-022-31112-x (PMC9203530; doi:10.1038/s41467-022-31112-x)
Supplement: Supplementary file 3 — Reporting Summary [file 41467_2022_31112_MOESM3_ESM.pdf]

Corresponding author(s): Changming Chen, Bihao Cao, and J.J. Emerson

Last updated by author(s): May 22, 2022

Reporting Summary

Nature Portfolio wishes to improve the reproducibility of the work that we publish. This form provides structure for consistency and transparency in reporting. For further information on Nature Portfolio policies, see our Editorial Policies and the Editorial Policy Checklist.

Statistics

For all statistical analyses, confirm that the following items are present in the figure legend, table legend, main text, or Methods section.

n/a	Confirmed	
☐	☒	The exact sample size (*n*) for each experimental group/condition, given as a discrete number and unit of measurement
☐	☒	A statement on whether measurements were taken from distinct samples or whether the same sample was measured repeatedly
☐	☒	The statistical test(s) used AND whether they are one- or two-sided<br>*Only common tests should be described solely by name; describe more complex techniques in the Methods section.*
☒	☐	A description of all covariates tested
☒	☐	A description of any assumptions or corrections, such as tests of normality and adjustment for multiple comparisons
☐	☒	A full description of the statistical parameters including central tendency (e.g. means) or other basic estimates (e.g. regression coefficient) AND variation (e.g. standard deviation) or associated estimates of uncertainty (e.g. confidence intervals)
☐	☒	For null hypothesis testing, the test statistic (e.g. *F*, *t*, *r*) with confidence intervals, effect sizes, degrees of freedom and *P* value noted<br>*Give P values as exact values whenever suitable.*
☒	☐	For Bayesian analysis, information on the choice of priors and Markov chain Monte Carlo settings
☒	☐	For hierarchical and complex designs, identification of the appropriate level for tests and full reporting of outcomes
☐	☒	Estimates of effect sizes (e.g. Cohen's *d*, Pearson's *r*), indicating how they were calculated

Our web collection on statistics for biologists contains articles on many of the points above.

Software and code

Policy information about availability of computer code

Data collection	Long reads: PacBio SEQUEL II;<br>Short reads: MGI SEQ 2000, Illumina Novaseq 6000.
Data analysis	G.C.E 1.0.2 - kmer frequencey;<br>MECAT2 2.1 - PacBio reads error correction;<br>CANU 2.0 - PacBio reads trim and genome assembly;<br>Pilon 1.23 - genome bases correction;<br>Juicer 1.56 - contigs anchor;<br>Juicebox 1.11.08 - contigs anchor;<br>3D-DNA 180114 - contigs anchor;<br>BUSCO 3.0.2 - quanlity assessment;<br>Minimap2 2.17 - similar sequence finding;<br>PAFR 0.0.2 - Generate synteny plot;<br>SMRTLink 8 - Iso-seq reads process;<br>EDTA 1.9.6 - TE annotation;<br>MAKER pipeline 3.01.03 - genome annotation;<br>HISAT2 2.2.1 - RNAseq reads mapping;<br>StringTie 2.1.4 - reconstruct gtf file;<br>Trimmomatic 0.38 - short reads clean up;<br>HiCExplorer 3.53 - Hi-C Matrix Building;<br>3DChromatin-ReplicateQC toolkit 0.0.1 - generate QuASAR quality scores;<br>Bismaker 0.23.1 - WGBS sequence process;

BWA 0.7.17 - short reads mapping;
MACS2 2.2.7.1 - Chipseq peak calling;
Deeptools 3.3.0 - output bam coverage result;
axtChain 377 - build long genome alignment chains;
TopDom 0.0.2 - TAD calling;
Limma 3.46.0 - gene expression patterns analysis;

For manuscripts utilizing custom algorithms or software that are central to the research but not yet described in published literature, software must be made available to editors and reviewers. We strongly encourage code deposition in a community repository (e.g. GitHub). See the Nature Portfolio guidelines for submitting code & software for further information.

Data

Policy information about availability of data

All manuscripts must include a data availability statement. This statement should provide the following information, where applicable:

- Accession codes, unique identifiers, or web links for publicly available datasets
- A description of any restrictions on data availability
- For clinical datasets or third party data, please ensure that the statement adheres to our policy

The data that support the findings of this study are available within the paper and its Supplementary Information files. A reporting summary for this article is available as a Supplementary Information file. The raw sequence data, processed data, and genome assembly (CA59) have been deposited into CNGB Sequence Archive (CNSA) of China National GeneBank DataBase (CNGBdb) with accession number CNP0001129 and National Center for Biotechnology Information (NCBI) with project accession PRJNA788020. All accessions of published Hi-C data used in this study are listed in Supplementary Table 21. The source data underlying Figs. 1d, 2b-c, 3b,e,f, 4a-d, 5a-b, 6b,c,e,f, 7a-f, as well as Supplementary Figs. 7d, 8a-b, 10b-c, and 12a are provided as a Source Data file.

Field-specific reporting

Please select the one below that is the best fit for your research. If you are not sure, read the appropriate sections before making your selection.

[x] Life sciences [ ] Behavioural & social sciences [ ] Ecological, evolutionary & environmental sciences

For a reference copy of the document with all sections, see nature.com/documents/nr-reporting-summary-flat.pdf

Life sciences study design

All studies must disclose on these points even when the disclosure is negative.

Sample size	Samples for RNA-seq, Hi-C, bisuflite sequencing, and ChIP-seq experiments were obtained as follows: Five randomly chosen plants were harvested and pooled to achieve each biological replicate. For RNA-seq, three such biological replicates were used, while for the other protocols, 2 replicates were used. The sample size was chosen to conservatively identify shared annotation states. For PacBio methods (DNA sequencing and ISO-seq), replication was not carried out because the goals of assembly and annotation did not include making claims about the variation in such data.
Data exclusions	No data was excluded.
Replication	RNA-seq data: 3 biological replicates; Hi-C: 2 biological replicates; Bisulfite sequencing: 2 biological replicates; ChIP-seq: 2 biological replicates; For PacBio and ISO-seq sequencing: only one replication was used. All attempts of the above-mentioned experiments were successful.
Randomization	For each sample category (RNA-seq, Hi-C, bisulfite sequencing, and ChIP), an individual replicate was derived from independent pools of 5 plants. Thus, allocation of all replicate samples are independent of each other.
Blinding	Experiments were blinded and carried out by different coauthors or researchers

Reporting for specific materials, systems and methods

We require information from authors about some types of materials, experimental systems and methods used in many studies. Here, indicate whether each material, system or method listed is relevant to your study. If you are not sure if a list item applies to your research, read the appropriate section before selecting a response.

Materials & experimental systems

n/a	Involved in the study
☒	☐ Antibodies
☒	☐ Eukaryotic cell lines
☒	☐ Palaeontology and archaeology
☒	☐ Animals and other organisms
☒	☐ Human research participants
☒	☐ Clinical data
☒	☐ Dual use research of concern

Methods

n/a	Involved in the study
☐	☒ ChIP-seq
☒	☐ Flow cytometry
☒	☐ MRI-based neuroimaging

ChIP-seq

Data deposition

☒ Confirm that both raw and final processed data have been deposited in a public database such as GEO.

☒ Confirm that you have deposited or provided access to graph files (e.g. BED files) for the called peaks.

Data access links
May remain private before publication.

The ChIP-seq raw data have been deposited into CNGB Sequence Archive (CNSA) of China National GeneBank DataBase (CNGBdb) with accession number CNP0001129 and National Center for Biotechnology Information (NCBI) with project accession PRJNA788020.

Files in database submission

Raw sequence reads
YZ-H3K27me3-1input,
YZ-H3K27me3-1,
YZ-H3K27me3-2input,
YZ-H3K27me3-2,
YZ-H3K4me3-1input,
YZ-H3K4me3-1,
YZ-H3K4me3-2input,
YZ-H3K4me3-2,
YZ-H3K9me2-1input,
YZ-H3K9me2-1,
Z-H3K9me2-2input,
YZ-H3K9me2-2.

Genome browser session
(e.g. UCSC)

Not submitted.

Methodology

Replicates

YZ-H3K27me3 : 2 biological replicates;
YZ-H3K4me3 : 2 biological replicates;
YZ-H3K9me2 : 2 biological replicates.

Sequencing depth

The reads number of YZ-H3K27me3-1input, YZ-H3K27me3-1, YZ-H3K27me3-2input, YZ-H3K27me3-2, YZ-H3K4me3-1input, YZ-H3K4me3-1, YZ-H3K4me3-2input, YZ-H3K4me3-2, YZ-H3K9me2-1input, YZ-H3K9me2-1, YZ-H3K9me2-2input, YZ-H3K9me2-2 are 53.4M, 44.5M, 39.4M, 41.7M, 39.2M, 47.3M, 54.9M, 55.1M, 51.4M, 45.6M, 46.2M, 45.5M separately. Mapped unique reads rate are 90.20%, 90.15%, 90.19%, 90.15%, 90.11%, 91.91%, 90.20%, 91.84%, 90.18%, 91.60%, 90.07%, 91.62%, separately. All the samples above are paired.

Antibodies

Antibodies used in this work include:
anti-H3K4me3 (Abcam ab8580);
anti-H3K9me2 (Abcam ab1220);
anti-H3K27me3 (Millipore 07-499).

Peak calling parameters

```
macs2 callpeak -t $prefix.sort.bam -c ${prefix}input.sort.bam -f BAMPE -g 3e9 -n $prefix.contain_input -q 0.05   --shift -100 --extsize 200 --nomodel -B
```

Data quality

We visualized peak signals on genome browser for each dataset, suggesting high-quality of ChIP-seq data and sufficient for our analysis purpose. The number of peaks (PDR < 0.05, default parameter) are:
YZ-H3K27me3-1 : 38177;
YZ-H3K27me3-2 : 33316;
YZ-H3K4me3-1 : 34528;
YZ-H3K4me3-2 : 33968;
YZ-H3K9me2-1 : 19218;
YZ-H3K9me3-2 : 15109;

Software

BWA 0.7.17 - short reads mapping;
MACS2 2.2.7.1 - Chipseq peak calling;
Deeptools 3.3.0 - Estimation of ChIP-seq reads coverage from .bam files
